# Uncoupling VEGFA Functions in Arteriogenesis and Hematopoietic Stem Cell Specification

**DOI:** 10.1016/j.devcel.2012.12.004

**Published:** 2013-01-28

**Authors:** Amy Leung, Aldo Ciau-Uitz, Philip Pinheiro, Rui Monteiro, Jie Zuo, Paresh Vyas, Roger Patient, Catherine Porcher

**Affiliations:** 1 MRC Molecular Haematology Unit, Weatherall Institute of Molecular Medicine, John Radcliffe Hospital, Oxford University, OX3 9DS Oxford, UK

## Abstract

VEGFA signaling is critical for endothelial and hematopoietic stem cell (HSC) specification. However, blood defects resulting from perturbation of the VEGFA pathway are always accompanied by impaired vascular/arterial development. Because HSCs derive from arterial cells, it is unclear whether VEGFA directly contributes to HSC specification. This is an important question for our understanding of how HSCs are formed and for developing their production in vitro. Through knockdown of the regulator ETO2 in embryogenesis, we report a specific decrease in expression of medium/long *Vegfa* isoforms in somites. This leads to absence of *Notch1* expression and failure of HSC specification in the dorsal aorta (DA), independently of vessel formation and arterial specification. *Vegfa* hypomorphs and isoform-specific (medium/long) morphants not only recapitulate this phenotype but also demonstrate that VEGFA short isoform is sufficient for DA development. Therefore, sequential, isoform-specific VEGFA signaling successively induces the endothelial, arterial, and HSC programs in the DA.

## Graphical abstract

**Graphical Abstract:**

**Figure:**
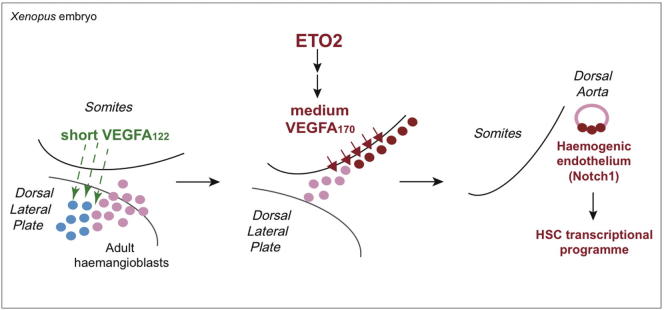

**Highlights:**

► VEGFA short isoform supports endothelial/arterial development of the dorsal aorta ► ETO2 maintains high levels of VEGFA medium and long isoforms in the somites ► VEGFA medium isoform instructs the blood program in blood stem cell precursors ► ETO2 specifies blood stem cells in a non-cell-autonomous manner

## Teaser

Leung et al. report that VEGFA short isoform is sufficient for endothelialization and arterialization of the dorsal aorta (DA); however, subsequent ETO2-dependent expression of VEGFA medium isoform in somites acts nonautonomously to instruct the hematopoietic stem cell (HSC) program in migrating DA/HSC precursors.

## Introduction

Defining the molecular mechanisms underlying stem cell specification is of interest from a developmental point of view and clinically relevant in regenerative medicine. In vertebrates, hematopoietic stem cells (HSCs) are generated from the endothelium of the ventral wall of the dorsal aorta (DA) in an evolutionarily conserved process (Dzierzak and Speck, 2008). Studies in *Xenopus* and zebrafish embryos have greatly contributed to the characterization of the earliest cellular, signaling, and transcriptional events that drive hematopoietic development (Ciau-Uitz et al., 2010a). In *Xenopus*, HSC production initiates with the development of adult hemangioblasts in the dorsal lateral plate (DLP) mesoderm. Progenitor cells then migrate to the midline of the embryo, where they form the DA, the arterial program is specified, and a subset of these endothelial cells is programmed toward a hematopoietic fate to form the hemogenic endothelium; hematopoietic cells (including HSCs) then bud out into circulation (Ciau-Uitz et al., 2010a).

During this process, multiple regulatory pathways work in concert to effect cell movement, tissue formation, and lineage specification. VEGFA is a signaling molecule with critical functions in embryonic vasculogenesis and angiogenesis (Carmeliet et al., 1996; Ferrara et al., 1996). It is required for the biology of endothelial cells as well as for arterial specification and, in that respect, is involved in multiple processes leading to HSC formation. VEGFA directs migration of the DA precursors toward the midline (Cleaver and Krieg, 1998). It is also required to trigger NOTCH signaling and the onset of the arterial program in endothelial cells (Hirashima, 2009; Lawson et al., 2002) and, more specifically, in the cells of the presumptive DA (Ciau-Uitz et al., 2010b; Gering and Patient, 2005), a process closely associated with the initiation of the HSC program in the hemogenic endothelium of the DA. Consequently, defects in the generation of HSCs are also observed on perturbation of VEGFA signaling (Ciau-Uitz et al., 2010b; Gering and Patient, 2005; Liang et al., 2001), when VEGFA receptor FLK1 is ablated (Shalaby et al., 1997) or when the NOTCH pathway is inhibited (Gering and Patient, 2005). So far, VEGFA has always been associated with HSC specification in the context of perturbed vasculogenesis or arterial specification; the complexity of VEGFA signaling has therefore made it difficult to address whether it has a direct role in HSC specification.

A number of transcription factors (TFs) play critical roles in association with signaling events in blood lineage specification and differentiation. ETO2, a member of the highly conserved ETO family of transcriptional corepressors (ETO/MTG8, ETO2/MTG16, and MTGR1; Davis et al., 2003), was originally identified through its involvement in a translocation breakpoint associated with acute myeloid leukemia (Gamou et al., 1998). It regulates the function of the hematopoietic TFs SCL and GATA1 during erythroid and megakaryocytic development (Goardon et al., 2006; Hamlett et al., 2008; Meier et al., 2006; Schuh et al., 2005). In addition, in a murine knockout, it is required for expansion of adult HSCs and multipotent progenitors in stress hematopoiesis conditions, as well as for normal fate of erythro-myeloid precursors (Chyla et al., 2008). ETO2 also controls NOTCH-dependent fate decisions in blood cells (Engel et al., 2010).

We have now characterized the function of ETO2 during HSC development in *Xenopus* embryos and linked it to major signaling pathways. We demonstrate that it is required at the onset of definitive HSC programming in a non-cell-autonomous manner. This function is mediated through the regulation of VEGFA expression in somitic tissue, prior to formation of the DA and specification of HSCs, at the time DA precursors migrate toward the midline. Through a combination of phenotypic rescue of ETO2-depleted embryos, generation of VEGFA hypomorphs, and isoform-specific knockdown of VEGFA, we have uncovered a specific function of VEGFA medium and/or long isoforms in HSC specification, independent of VEGFA requirement in vasculogenesis and arterial specification. This activity is essential for *Notch1* expression in the hemogenic endothelium and for the onset of the hematopoietic transcriptional program. Separately, we show that, prior to HSC specification, VEGFA short isoform is sufficient for endothelialization and arterialization of the DA. By uncoupling the different activities of VEGFA during endothelial and hematopoietic development, our model reveals an ETO2/VEGFA/NOTCH1 regulatory cascade that initiates in the somites and directly controls HSC specification.

## Results

### ETO2 Knockdown Blocks Specification of Definitive Hematopoiesis in *Xenopus*

To study ETO2 function in HSC development, we exploited the anatomical and experimental advantages of the *Xenopus* model, in particular the access to HSC precursors at an early stage (Ciau-Uitz et al., 2010a). In *Xenopus laevis*, genes are present in two “pseudoallele” forms. We designed two antisense morpholinos (MOs) that knockdown *Eto2* expression from both alleles: *Eto2*-MO was directed against the translation initiation ATG, and *Eto2*-MO2 was directed against the 5′ UTR (Figures S1A and S1B available online).

**Figure 1:**
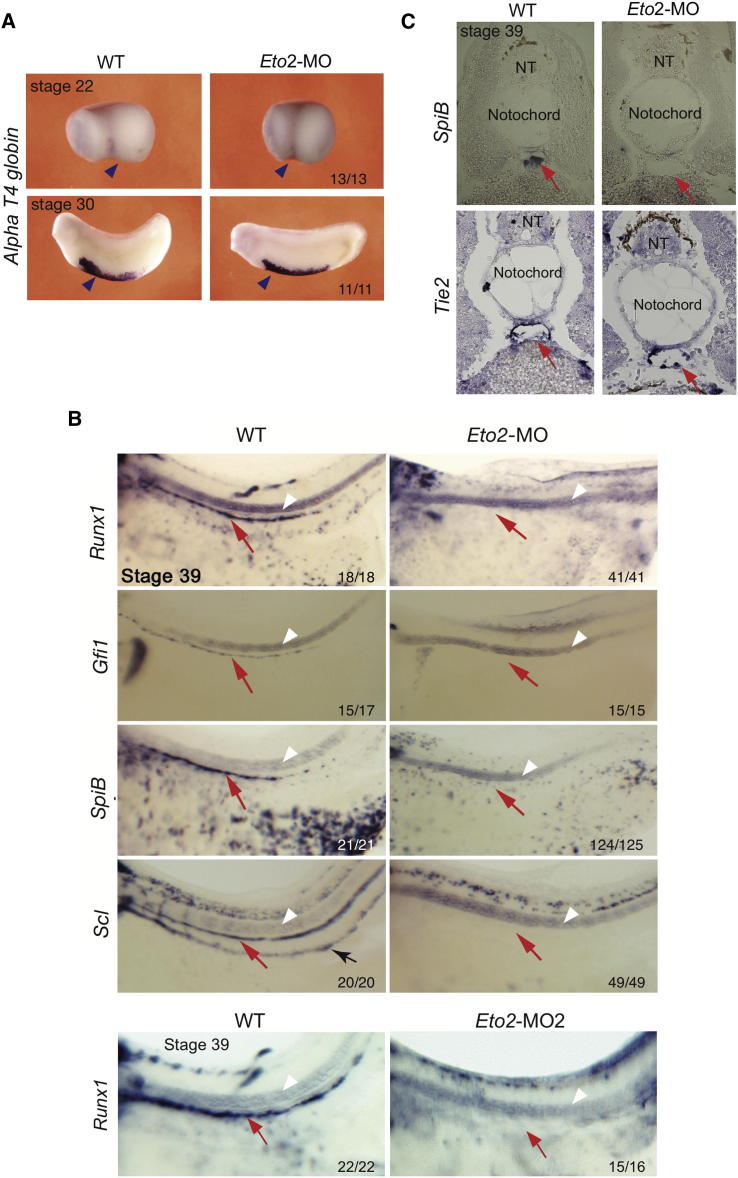
ETO2 Is Necessary for the Onset of Definitive Hematopoiesis (A) Expression analysis showing no difference in expression of primitive erythroid gene *alpha T4 globin* at stages 22 and 30 in WT and *Eto2* morphant embryos. Blue arrowheads, VBI. (B and C) ETO2 morphants show absence of expression of *Runx1*, *Gfi1*, *SpiB*, and *Scl* in the DA (red arrows) at stage 39. (B) Whole-mount in situ hybridization, WMISH. White arrowheads indicate the notochord. The black arrow (*Scl* panel, WT embryo) indicates the posterior cardinal vein (PCV) containing *Scl*-positive primitive erythrocytes. Staining is absent in the ETO2 morphants because circulation is delayed. (C) In situ hybridization on section (ISHS) for *SpiB* and the endothelial marker *Tie2*; note that the DA has formed and is lumenized in *Eto2* morphants. NT, neural tube. Numbers in the bottom of the panels denote the number of embryos as shown in the panel out of the total examined. Whole mounts are shown with anterior to the left and dorsal to the top. Sections are in transverse orientation with dorsal to the top. See also Figures S1 and S4.

Hematopoiesis was examined at various stages of development in *Eto2* morphant embryos. In *Xenopus*, primitive erythroid cells are generated in the ventral blood islands (VBI), a site analogous to the murine yolk sac. The VBI start to form at stage 22 on the ventral side of the embryo and continue to develop through stage 30 (Kau and Turpen, 1983). Expression of the primitive erythroid marker *alpha T4 globin* at stages 22 and 30 was unaffected in *Eto2* morphants (Figure 1A). Ablation of *Eto2* expression therefore did not perturb the initiation or differentiation of the primitive erythroid lineage.

A second wave of hematopoiesis during embryogenesis gives rise to the definitive (adult) blood lineage emanating from HSCs. In *Xenopus*, specification of the endothelium of the ventral wall of the DA toward the adult hematopoietic lineage can be assessed by stage 39 of development by the expression of hematopoietic markers (Figure 1B; Ciau-Uitz et al., 2010a). In contrast, in *Eto2* morphants (*Eto2*-MO and *Eto2*-MO2) there was a complete absence of the key hemogenic endothelium and HSC TF, *Runx1*, as well as the other HSC markers *SpiB (Pu.1* homolog), *Gfi1*, and *Scl* in the DA (Figures 1B, 1C, and S1C). Although expression of hematopoietic markers was not detected in the DA, the vessel had formed, was endothelialized and lumenized (Figure 1C, *Tie2* sections).

Therefore, ablation of ETO2 expression during *Xenopus* embryogenesis leads to a specific block in the definitive HSC program in the DA.

### ETO2 Is Not Required for Specification of the Arterial Program in the DA or the Adult Hemangioblast

To further characterize the defects in HSC specification, we analyzed *Eto2* morphants at earlier stages of development, before specification of the hematopoietic fate in the endothelium of the DA.

**Figure 2:**
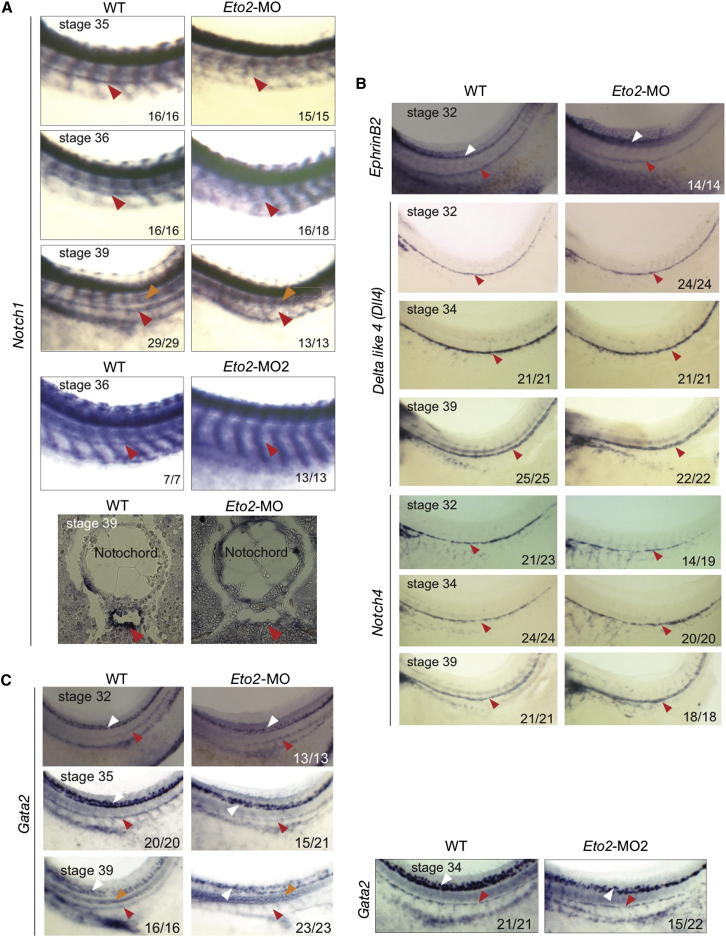
ETO2 Is Specifically Required for the Establishment of the Hematopoietic Program in the DA (A) *Notch1* is absent in the DA of *Eto2*-MO and *Eto2*-MO2 morphants (stages 35/36/39, WMISH, and stage 39 bottom, ISHS; red arrowheads, DA; orange arrowheads, notochord). (B and C) Expression analysis of arterial-affiliated genes *EphrinB2*, *Delta-like4* (*Dll4*), and *Notch4* (B), as well as *Gata2* (C) in the presumptive DA (stage 32) and in the DA (stages 34–39) in *Eto2* morphants, red arrowheads. White and orange arrowheads: neural tube and notochord, respectively. Numbers in the bottom of the panels denote the number of embryos as shown in the panel out of the total examined. Whole mounts are shown with anterior to the left and dorsal to the top. Sections are in transverse orientation with dorsal to the top. See also Figures S2A and S4.

One critical regulator of HSC specification in the hemogenic endothelium is NOTCH1 (Kumano et al., 2003). In *Xenopus*, its expression is first detected in the DA at stage 34, preceding that of blood genes, such as *Runx1*, that initiates at stage 35 in the ventral wall of the DA (A.C.-U. and R.P., unpublished data). In *Eto2* morphants (*Eto2*-MO and *Eto2*-MO2), *Notch1* expression was absent within the DA at stages 35, 36, and 39 (Figure 2A). Therefore, *Notch1* expression is not specified, and the aberrant programming of the HSC population is already apparent at stage 35 in *Eto2* morphants.

Before the onset of the HSC program in the DA, the vessel is specified as an artery. We examined expression of the arterial markers *EphrinB2*, *Delta-like 4* (*Dll4*), *Notch4*, and *Cx37* in the DA of *Eto2* morphants from stages 32 to 39. Expression was unaffected in the presumptive DA at stage 32 (*EphrinB2*, *Dll4*, and *Notch4*; Figure 2B) and was maintained at later stages of development in the DA (*Dll4*, *Notch4*, and *Cx37*; Figures 2B and S2A, stages 34/39), suggesting that ETO2 is required neither for the initiation nor the maintenance of the arterial program. Interestingly, the early hematopoietic target of NOTCH, *Gata2* (Robert-Moreno et al., 2005), was detected in the midline in ETO2-depleted embryos at stage 32, but its expression was only present in approximately two-thirds of the morphants analyzed at stages 34 (*Eto2*-MO2) and 35 (*Eto2*-MO) and was absent at stage 39 (Figure 2C, red arrowheads).

**Figure 3:**
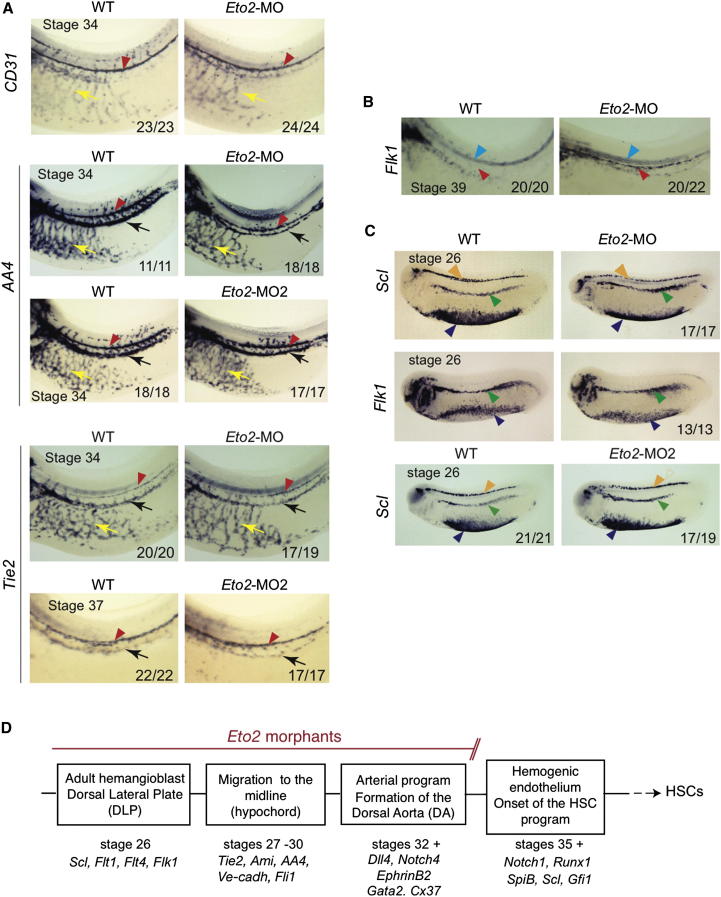
The Major Axial Vessels Are Normal and Adult Hemangioblasts Are Specified in ETO2 Morphants (A) The endothelial markers *CD31*, *AA4*, and *Tie2* are expressed normally in the axial vessels of *Eto2* morphants (*Eto2*-MO and *Eto2*-MO2), revealing the DA (red arrowhead), the PCV (black arrow), and the trunk vasculature (yellow arrow). (B) Abnormal expression of *Flk1* in *Eto2* morphant DA at stage 39 (red arrowhead), as a consequence of the absence of *Notch1*. Blue arrowhead: notochord. (C) Specification of adult hemangioblasts is unaffected in ETO2-deficient embryos. *Scl* is expressed normally at stages 26 in the DLP, primitive erythroid cells, and neurons in *Eto2* morphants (*Eto2-*MO and *Eto2-*MO2, green, blue, and orange arrowheads, respectively). *Flk1* is also expressed normally in the DLP and trunk endothelium of *Eto2* morphants (green and blue arrowheads, respectively). Numbers in the bottom right of the panels denote the number of embryos as shown in the panel out of the total examined. Whole mounts are shown with anterior to the left and dorsal to the top. Sections are in transverse orientation with dorsal to the top. (D) Schematic diagram depicting the succession of events leading to HSC specification and the requirement for ETO2. The earliest defect in *Eto2* morphants is the loss of *Notch1* expression. Molecular markers analyzed in this study are indicated for each stage of development shown. See also Figures S2B–S2D and S4.

Hematopoiesis and vascular development are closely linked. To further document vessel development in *Eto2* morphants, we examined expression of several endothelial markers. We show normal expression of *VE-cadh* and *Ami* (stage 37), *CD31*, *AA4*, *Tie2*, and *Fli1* (stage 34) in *Eto2* morphants, confirming formation of the DA as an endothelium and showing normal trunk vasculature (Figures 3A and S2B, red arrowhead and yellow arrow, respectively). *AA4*, *Tie2*, *Ami*, and *Fli1* are also expressed in the posterior cardinal vein (PCV, Figures 3A and S2B, black arrow), providing additional support for a largely normal vasculature in *Eto2* morphants. Two interesting observations emerged from our analysis of the vasculature. First, *AA4* staining in uncleared embryos at stage 34 revealed that intersomitic vessel (ISV) sprouting from the PCV was greatly impaired in *Eto2* morphants (Figure S2C), suggesting a specific function of ETO2 in this process. Second, *Flk1* expression, normally repressed by NOTCH1 signaling (Taylor et al., 2002), persisted in the DA of the morphants at stage 39 (Figure 3B), consistent with the absence of *Notch1*.

The DA and its derivatives, including the HSCs, derive from adult hemangioblasts located in the DLP in early embryogenesis (Ciau-Uitz et al., 2010a; Walmsley et al., 2002). To monitor specification of these cells, we examined expression of the hematopoietic and endothelial markers, *Scl*, *Flk1*, *Flt1*, and *Flt4*, at stage 26. All markers were normally expressed in *Eto2* morphant DLP (Figures 3C and S2D, green arrowheads). ETO2 is therefore not required for the specification of the DA/HSC precursors in the DLP.

In summary, ETO2 is not required for programming the earliest DA precursors and is dispensable for vessel formation and specification as endothelia. Moreover, it is not necessary for the establishment or the maintenance of the arterial program in the DA. The first molecular defect detected in *Eto2* morphants is the loss of *Notch1* expression at stage 35, concomitant with a decrease in expression of *Gata2*. ETO2 is therefore required at the onset of the HSC program for development of the hemogenic endothelium (Figure 3D).

### A Non-Cell-Autonomous Role for ETO2 in HSC Specification

To link *Eto2* expression to the phenotype observed in *Eto2* morphants, we examined its pattern of expression during hematopoietic development. We only present expression of one of the two pseudoalleles (A), as the expression patterns of both are identical.

**Figure 4:**
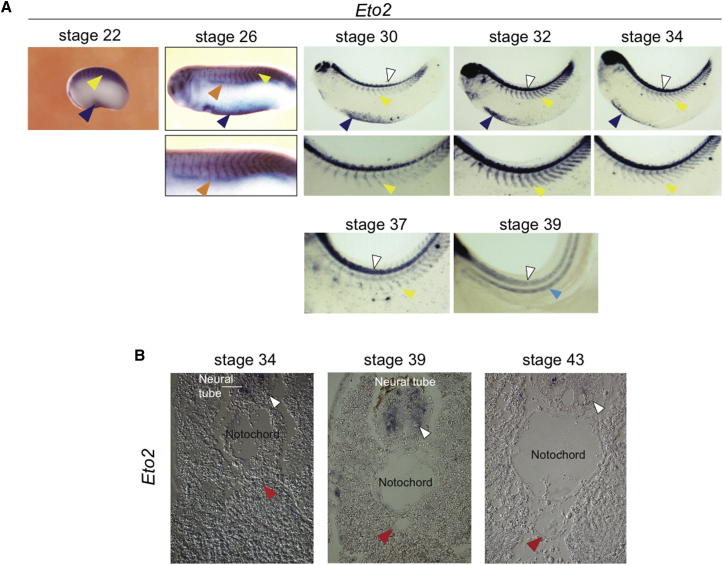
*Eto2* Is Expressed in Precursors of Primitive but Not Definitive Blood (A) *Eto2* expression (WMISH) from stage 22 to stage 39. *Eto2* is found in somitic tissues (yellow arrowheads), the developing VBI (dark blue arrowheads, up to stage 34), and, from stage 30, the neural tube (white arrowheads). At stage 26, no expression was detected in the DLP, delineated by *Lmo2* expression (orange arrowhead). Light blue arrowhead, notochord. (B) *Eto2* expression (ISHS) was not seen in the DA (red arrowheads, stages 34–43). Whole mounts are shown with anterior to the left and dorsal to the top. Sections are in transverse orientation with dorsal to the top. See also Figures S3 and S4.

In early development, *Eto2* expression is detected in primitive blood in the VBI and in the somites (Figure 4A, stages 22 and 26, blue and yellow arrowheads, respectively). As *Eto2* morphants exhibit no gross defects with respect to primitive blood development (see *Scl* and *globin* expression, Figures 1A and 3C), ETO2 is not critically required for specification of this lineage. In these early stages, *Eto2* expression is not seen in the DLP (Figure 4A, stage 26, orange arrowheads). As development proceeds, its expression decreases in the somites and is no longer detected in this tissue by stage 39 (Figure 4A, yellow arrowheads). Importantly, *Eto2* could not be detected in the presumptive DA (stage 34), in the DA itself after it has lumenized (stage 39), or when definitive hematopoietic cells, including HSCs, are found associated with the ventral wall of the DA (stage 43) (Figure 4B, red arrowheads). *Eto2* is also detected in the neural tube throughout development (Figures 4A and 4B, white arrowheads). Absence of *Eto2* in the DA was confirmed by laser capture microdissection followed by RT-qPCR (Figure S3A).

Expression of *Eto*-related transcripts was examined in parallel; *Eto*, *Mtgr1*, and two transcripts highly similar to *Mtgr1* (*Mtgr1-like1* and *Mtgr1-like2*) showed no overlap with expression of *Eto2* and no expression in hematopoietic tissues at the stages of development that were examined (except for a faint staining for *Mtgr1-like2* at stage 43 in DA, Figures S3B and S3C). Importantly, the MOs used in this study are not predicted to target the *Eto*-related transcripts (Figure S1A).

In summary, *Eto2* expression is not detected in HSC/progenitor cells, in the DA, or their precursors at any stage of development. Moreover, *Eto2* pattern of expression in zebrafish is similar to that described in *Xenopus* (Figures S4A–S4C); in addition, and extending previous data (Meier et al., 2006), depletion of *Eto2* in zebrafish reveals a phenotype similar to the one described in *Xenopus* and in agreement with a function for ETO2 in HSC specification (Figures S4D and S4E). In conclusion, loss of the HSC program upon knockdown of ETO2 is likely to occur through a non-cell-autonomous mechanism.

### ETO2 Regulates *Vegfa* Expression in the Somites

The developing somites are in close physical proximity to the DLP, the migration path of the DA/HSC progenitors, and the hypochord, site of DA formation. *Eto2* expression initiates very early during somitic development (stage 22), is at its highest at stages 26/27 and then gradually decreases (Figure 4A). Because no *Eto2* expression was detected in the DA/HSC or their precursors, we hypothesized that somitic expression of ETO2 might be required for HSC formation.

In order to understand how ETO2 might regulate the HSC program from the somites, we assessed gene expression in the somites of stage 27 *Eto2* morphants. We monitored expression of genes involved in signaling pathways (*Shh*, *Notch1*, and *Nrp1*) or coding for TFs (the NOTCH target gene *Hesr1* as well as *Hif1α*, *Arnt*), necessary for the correct development of the somites and known regulators of endothelial cell and/or HSC development at this early developmental stage (Diez et al., 2007). None of these genes were affected (Figure S5).

**Figure 5:**
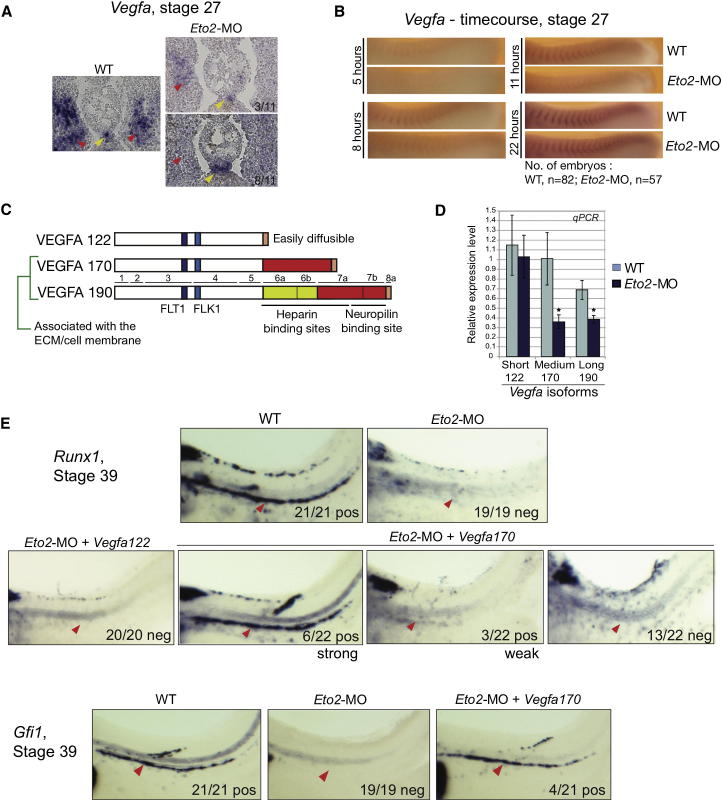
ETO2 Regulates HSC Development through VEGFA_170_ (A) *Vegfa* expression is downregulated (top right panel) or absent (bottom right panel) in the somites of stage 27 *Eto2* morphants (red arrowheads) but maintained in the hypochord (yellow arrowheads), as revealed by ISHS (transverse sections with dorsal to the top). (B) WMISH staining time course reveals downregulation of *Vegfa* in *Eto2* morphant somites; the staining is markedly lighter in morphant embryos at 8 and 11 hr after addition of the staining substrate (anterior to the left, dorsal to the top). (C) Alternative splicing generates VEGFA isoforms with different biological properties. In *X. laevis*, there are three known VEGFA isoforms (VEGF_122_, VEGFA_170_, and VEGFA_190_). FLK1, FLT1: binding domains for receptors. The C-terminal domain of isoforms VEGFA_170_ and VEGFA_190_ is responsible for association with the extracellular matrix (ECM) and the cell membrane. VEGF_122_ is the diffusible isoform (Cleaver and Krieg, 1998). (D) Levels of isoform-specific VEGFA transcripts in WT and *Eto2* morphant somite-hypochord regions at stage 27 (n = 5) quantitated by qPCR. Error bars denote SEM. ^∗^p < 0.05. (E) Exogenous *Vegfa*_170_ expression rescues the HSC program in *Eto2* morphants. Embryos were coinjected with *Eto2*-MO only or *Eto2*-MO and 4 ng of mRNAs encoding *Vegfa*_*122*_*or Vegfa*_*170*_ isoforms. Rescue of *Runx1* and *Gfi1* expression in DA (WMISH, red arrowheads) was observed only with V*egfa*_170_ mRNA. Pos, positive; neg, negative. Whole mounts are shown with anterior to the left; dorsal at the top. See also Figure S5.

Some of our phenotypic observations suggested that VEGFA, closely involved in processes associated with HSC formation, might function downstream of ETO2. Indeed, intersomitic vessel sprouting from the PCV, a process regulated by VEGFA signaling from the somites (Kälin et al., 2007), is perturbed in *Eto2* morphants (Figures S2C). We therefore examined *Vegfa* expression in stage 27 embryos. *Vegfa* is strongly expressed in the somites and the hypochord of WT embryos (Figure 5A, left panel, red and yellow arrowheads, respectively). In *Eto2* morphants, partial downregulation of *Vegfa* was specifically observed in the somites, whereas expression was unchanged within the hypochord (Figure 5A, right panels). The partial nature of the downregulation was confirmed by a staining time course; a decrease in the intensity of expression in the somites of *Eto2* morphants was particularly striking at 8 and 11 hr of staining (Figure 5B).

VEGFA exists in three main isoforms generated by alternative splicing (in *Xenopus*, short VEGFA_122_, medium VEGFA_170_, and long VEGFA_190_) that differ in their physical and biological properties (Figure 5C). The short isoform lacks the extracellular matrix (ECM)-binding domains (heparin and neuropilin binding sites) and is the most diffusible form of the protein. The long isoform is tethered to the ECM by the full complement of domains that mediate strong interaction with heparin. The medium isoform only contains part of the heparin-interaction sequences, and only 50%–70% of VEGFA_170_ binds to the cell/ECM (Robinson and Stringer, 2001). The isoforms are differentially expressed in somites and the hypochord of *Xenopus* embryos; VEGFA_122_ is the predominant isoform in the hypochord, whereas all three isoforms are found in somitic tissue (Cleaver and Krieg, 1998).

Real-time PCR analysis was carried out to determine the levels of the different *Vegfa* isoforms in somite/hypochord regions of stage 27 WT and *Eto2* morphant embryos. Levels of *Vegfa*_190_ and, more so, *Vegfa*_170_ were decreased in *Eto2* morphants, in contrast to *Vegfa*_122_, which remained largely unaffected (Figure 5D). The specific downregulation of the two larger *Vegfa* isoforms is in agreement with the visual observation (by ISHS) that downregulation of *Vegfa* in the *Eto2* morphants is limited to somitic tissue. Whether *Vegfa*_122_ is also downregulated in the somites is difficult to judge, as the hypochord region had to be included in the PCR analysis. A detectable decrease in the level of this isoform within the somites may therefore have been masked by its continued expression within the hypochord in *Eto2* morphants.

ETO2 expression therefore is required to establish correct levels of *Vegfa*_*170*_ and *Vegfa*_*190*_ in the somites at stage 27, when precursors of the DA and HSCs are starting to migrate toward the midline.

### The *Eto2* Morphant Phenotype Is Rescued by Exogenous Expression of *Vegfa*_*170*_ Isoform

Next, we asked whether downregulation of *Vegfa* could be responsible for the loss of the HSC program in the *Eto2* morphants by performing rescue experiments.

*Eto2*-MO was coinjected with various concentrations of messenger RNAs (mRNAs) encoding the different VEGFA isoforms. The embryos were harvested at stage 39 and examined for expression of the HSC marker, *Runx1*, to assess the hematopoietic program. No rescue was observed with 2 or 3 ng *Vegfa* mRNA (short, medium, and long; data not shown). In contrast, 9/22 embryos coinjected with *Eto2*-MO and 4ng *Vegfa*_*170*_ mRNA exhibited *Runx1* expression in the DA, six of them showing strong rescue; 4/21 embryos also showed rescue of *Gfi1* expression (Figure 5E). Importantly, 4 ng *Vegfa*_*122*_ mRNA did not rescue *Runx1* expression (Figure 5E). At that concentration, *Vegfa*_*190*_ was toxic, and its effects could not be examined. As a control for *Vegfa* activity, ectopic expression of *Dll4* (a sensor of VEGFA levels [Coultas et al., 2010]) was observed in the vitelline vessels of all embryos coinjected with *Vegfa* isoforms (data not shown). In conclusion, restoration of high levels of *Vegfa*_*170*_, but not *Vegfa*_*122*_, was sufficient to re-establish the HSC program.

### *Vegfa* Hypomorphs Present Similarities with the *Eto2* Morphants

**Figure 6:**
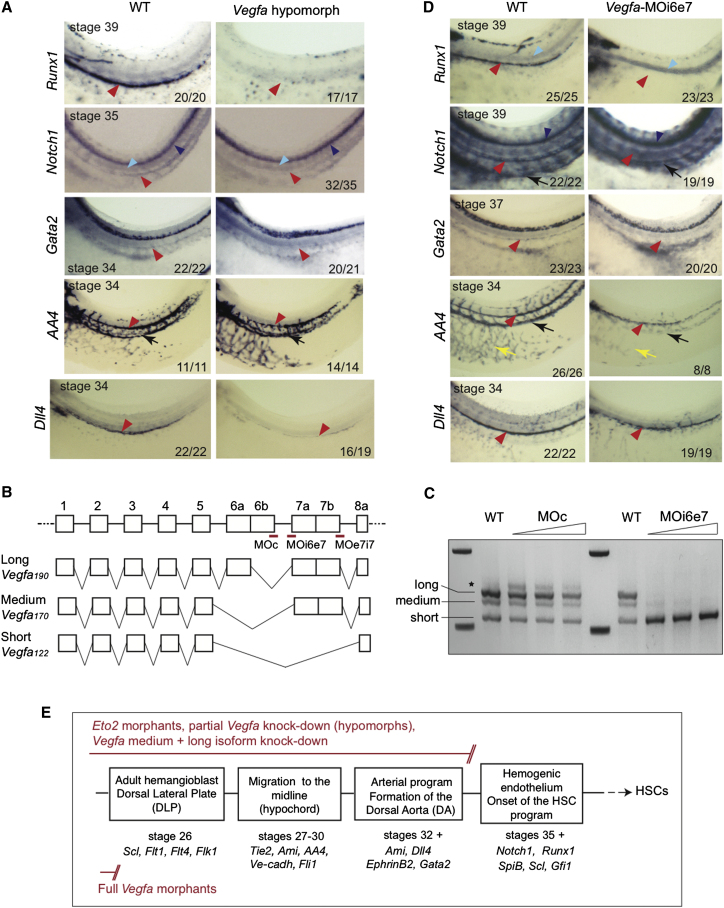
Hypomorphic and Isoform-Specific Knockdowns of *Vegfa* Phenocopy the *Eto2* Morphant Phenotype (A) Hypomorphic VEGFA phenotype. The HSC program was abrogated at stage 39 (loss of *Runx1* expression), and the early HSC markers *Notch1* and *Gata2* were absent at stages 34/35 in VEGFA hypomorphs. DA formation (as revealed by the endothelial marker *AA4*, at stage 34) is unaffected in VEGFA hypomorphs. Decreased expression of arterial marker *Dll4* is observed in the DA of *Vegfa* hypomorphs at stage 34. (B) Top: structure of *Xenopus laevis Vegfa* gene. Open boxes, exons; thin lines, introns; not to scale. Red lines show the position of the control MO (MOc) and the MOs targeting the intron6/exon7 (MOi6e7) and exon7/intron7 (MOe7i7) splice junctions. Depicted below are the splicing events giving rise to the three known *Vegfa* isoforms. (C) *Vegfa* short, medium, and long mRNA isoforms were detected by PCR from material isolated from stage 27 wild-type (WT) embryos or embryos injected with increasing concentrations of MOi6e7 or control MOc (20, 30, and 40 ng). The ethidium-bromide-stained gel shows knockdown of expression of medium and long mRNA isoforms and increased production of the short isoform in MOi6e7 morphants. Asterisk: the band may represent one of the minor *Vegfa* isoforms as detected in human and mouse, although this has not been confirmed by sequencing. (D) *Vegfa* medium and long isoform morphant phenotype (*Vegfa*-MOi6e7). As for *Vegfa* hypomorphs (A), expression of *Runx1*, *Notch1*, and *Gata2* is lost. DA formation is unaffected, as revealed by *AA4* expression at stage 34. In contrast to *Vegfa* hypomorphs, expression of the arterial marker *Dll4* appears normal. Red arrowheads, DA; light blue arrowheads, notochord; dark blue arrowheads, neural tube; black arrows, posterior cardinal veins (PCV); yellow arrows, trunk vasculature. Numbers at the bottom of the panels indicate the number of embryos with the given phenotype out of the total number examined. Whole mounts are shown with anterior to the left and dorsal to the top. (E) Schematic diagram detailing the phenotypes of ETO2 morphants, VEGFA hypomorphs, and VEGFA morphants with respect to the definitive HSC program. Molecular markers analyzed in this study are indicated for each stage of development shown. See also Figure S6.

To confirm the importance of VEFGA in HSC specification, we tried to phenocopy the *Eto2*-morphant phenotype by partially downregulating *Vegfa*. To achieve this, we injected a *Vegfa* MO that targets all isoforms (Kälin et al., 2007). Full knockdown of VEGFA with this MO ablates the correct specification of the definitive blood precursors in the DLP, prevents their migration to the midline and, consequently, precludes DA formation (Ciau-Uitz et al., 2010b). A phenotype similar to that observed in ETO2-depleted embryos was achieved by injecting half the amount of *Vegfa* MO (*Vegf*-MO, 12.5 ng per embryo instead of 25 ng for full knockdown). This ablated the HSC program in the DA, without compromising DA formation. Indeed, *Vegfa* hypomorphs expressed neither the HSC gene, *Runx1*, nor the early hematopoietic markers *Gata2* and *Notch1* in the DA (Figure 6A). DA formation was unaffected (*AA4* and *Ami,* stages 34/37; Figures 6A and S6A); note the defect in ISV sprouting (Figure S6A, *AA4*, uncleared embryos), as observed in *Eto2* morphants (Figure S2C). In contrast to the *Eto2* morphants, expression of the arterial markers *Dll4* and *Cx37* was weak when compared to wild-type controls (Figure 6A and S6A, stages 34/35). Finally, the DLP continues to express *Scl*, *Flk1*, and *Flt4* in the *Vegfa* hypomorphs (Figures S6A), as seen for the *Eto2* morphants (Figures 3C and S2D) and contrary to *Vegfa* full knockdown embryos (Ciau-Uitz et al., 2010b) (Figure 6E).

Therefore, there are clear parallels between the phenotypes observed in *Eto2* morphants and *Vegfa* hypomorph embryos: absence of expression of HSC markers, specification of the arterial program, and normal endothelialization. Expression of arterial genes in *Vegfa* hypomorphs was however decreased, suggesting the existence of a critical threshold in the levels of VEGFA isoforms (or at least one of them) to fully support arterial development.

### Isoform-Specific Knockdown of *Vegfa*_*170*_ and *Vegfa*_*190*_ Fully Phenocopies the *Eto2* Morphant Phenotype in the DA

To firmly establish the functions of VEGFA individual isoforms in endothelial, arterial, and HSC specification, we designed isoform-specific MOs. We isolated *X. laevis Vegfa* genomic sequences from raw sequence data kindly provided by Richard Harland (University of California, Berkley) and characterized the exon-intron boundaries (Figure 6B). We identified eight exons, as previously described (1 to 8a, Figures 5C and 6B) (Nowak et al., 2008; Xu et al., 2011) and focused on the exon/intron boundaries involved in alternative splicing (exons 6 and 7, Figure 6B). Because of the way the short and medium isoforms are produced, it was not possible to design MOs that would specifically target either of them. Blocking the intron5/exon6 acceptor splice site is predicted to only interfere with production of the long isoform; however, the sequences were not suitable for MO design. We therefore intended to specifically target the medium and long, anchored isoforms. To do this, we designed two MOs targeting splice junctions involved in production of both medium and long isoforms (junction intron6/exon7: *Vegfa*-MOi6e7, and junction exon7/intron7: *Vegfa*-MOe7i7, Figure 6B). Both MOs produced similar data; we only present those obtained with *Vegfa*-MOi6e7. To confirm correct targeting, increasing concentrations of *Vegfa*-MOi6e7 were injected into embryos and expression of *Vegfa* mRNA isoforms detected by PCR at stage 27 (with primers recognizing all isoforms, *Vegfa* FL, Supplemental Experimental Procedures). PCR products were analyzed by electrophoresis (Figure 6C) and sequencing (data not showm), confirming the identity of the isoforms. The long mRNA isoform was absent in *Vegfa*-MOi6e7 morphants. Formation of the medium mRNA isoform was absent at MO concentrations >20 ng and not detectable by sequencing. Production of the short isoform increased with increasing concentrations of the MO. Injection of a control MO directed at a splice junction not involved in isoform production (MOc) did not affect production of *Vegfa* short, medium, and long mRNAs. We reasoned that *Vegfa*-MOi6e7 would allow the distinction between VEGFA functions mediated by the short, diffusible isoform and those mediated by the medium and long, anchored isoforms.

We injected *Vegfa*-MOi6e7 and monitored endothelialization, arterialization, and hematopoiesis in developing morphants. Strikingly, knockdown of *Vegfa* medium and long mRNA fully recapitulated the *Eto2* morphant phenotype in hematopoietic development; expression of *Vegfa*_*122*_ was sufficient to specify the endothelial and arterial programmes of the DA but failed to support HSC specification, thereby confirming the specific requirement of long/medium isoforms in this process. Indeed, the *Vegfa*-MOi6e7 morphants showed no detectable expression of HSC-affiliated genes (*Runx1*, *Gfi1*, stage 39, Figures 6D and S6B) and early hematopoietic markers (*Notch1*, *Gata2*, stages 39/37, Figures 6D and S6B), but we observed normal expression of genes associated with the endothelial (*AA4*, *Flk1*, *CD31*, stages 34/37, Figures 6D and S6B) and arterial (*Dll4*, *Cx37*, *Notch4*, stages 34/39, Figures 6D and S6B) programs of the DA, as well as with adult hemangioblasts in the DLP (*Scl*, *Flk1*, stage 26, Figure S6B). Note the absence of ISV sprouting, as for *Eto2* morphants and *Vegfa* hypomorphs (*AA4*, Figure S6B). We also noticed limited development of PCV and the trunk vasculature in the morphants (Figures 6D and S6B, black and yellow arrows, respectively), suggesting that low/intermediate levels of medium and/or long *Vegfa* isoforms are likely to be required for this process.

In conclusion, by partially perturbing the VEGFA pathway, indirectly through downregulation of ETO2 in somitic tissue and directly through VEGFA knockdown approaches, our study reveals an isoform-specific function of VEGFA at the onset of the HSC program, independent from its functions in formation and specification of the DA and mediated by the medium and/or long isoforms. It also highlights that VEGFA_122_ activity is sufficient to support processes prior to HSC specification, i.e., hemangioblast specification, DA precursor migration, vessel formation, and arterialization of the DA.

### No Cross-Regulation between ETO2, ETV6, and Hedgehog

Studies of signaling pathways and TFs acting upstream of HSC specification have highlighted regulatory cascades between the TF ETV6 and VEGFA in *Xenopus* (Ciau-Uitz et al., 2010b) and between Hedgehog (HH) signaling pathway and VEGFA in zebrafish but not in mouse (Coultas et al., 2010; Gering and Patient, 2005; Lawson et al., 2002). We set out to define possible epistatic relationships between these regulatory pathways and ETO2 in the somites of stage 26 *Xenopus* embryos.

**Figure 7:**
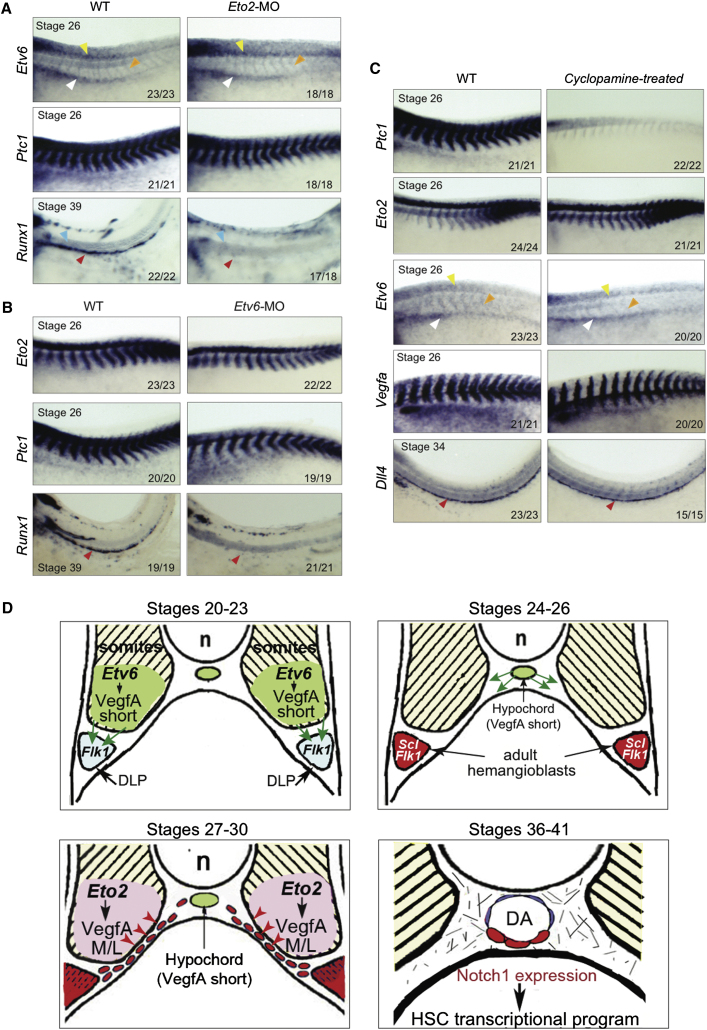
No Epistasis between ETO2, ETV6, and Hedgehog Signaling (A–C) Expression analysis by WMISH of *Etv6*, *Eto2*, *Ptc1*, *Vegfa*, and *Dll4* in *Eto2* (A) and *Etv6* (B) morphants as well as in embryos treated with cyclopamine (C). Efficiency of *Eto2* and *Etv6* morpholinos is controlled by *Runx1* staining at stage 39, and that of cyclopamine is controlled by *Ptc1* staining. Red arrowheads, DA; orange arrowheads, *Etv6* staining in the somites; blue arrowheads, notochord; yellow arrowheads, neural tube; white arrowheads, DLP. Numbers at the bottom of the panels indicate the number of embryos with the given phenotype out of the total number examined. Whole mounts are shown with anterior to the left and dorsal to the top. (D) Uncoupling the requirement for VEGFA isoforms during HSC development in *Xenopus*. Schematic diagram depicting the requirements for VEGFA isoforms in the succession of events leading to HSC formation. Stages 20–23: ETV6 regulates production of VegfA in the somites, VegfA short isoform is critical for correct programming of the adult hemangioblasts in the DLP (green arrows). Stages 24–26: the adult hemangioblasts (or DA precursors) express both endothelial (*Flk1*) and hematopoietic (*Scl*) markers and *Vegfa* in an autocrine manner. The hypochord secretes VegfA short isoform that guides migration of hemangioblasts to the midline (green arrows). Stages 27–30: ETO2 regulates production of VegfA long/medium isoforms (VegfA M/L) in the somites. These isoforms instruct the hematopoietic program of the DA precursors as they migrate along the somites (red arrowheads). Stages 36–41: the DA has formed and is specified as an artery. Cells in the hemogenic endothelium express NOTCH1; this will trigger expression of the HSC transcriptional program. n, notochord.

In *Eto2* morphants, *Etv6* somitic expression was not affected (Figure 7A, orange arrowheads). To examine the HH pathway, we analyzed expression of *Ptc1*, an HH receptor that serves as a readout for HH activity (Perron et al., 2003); its strong somitic expression was not perturbed in *Eto2* morphants (Figure 7A). Similarly, *Etv6* knockdown affected neither *Eto2* somitic expression nor HH signaling (*Eto2* and *Ptc1*, Figure 7B). Cyclopamine-mediated inhibition of HH signaling (Perron et al., 2003) showed that, although *Ptc1* expression is completely dependent on HH, *Eto2* and *Etv6* are regulated independently of the HH pathway as their somitic expression in cyclopamine-treated embryos was similar to that in wild-type embryos (Figure 7C). Unlike what has been described in zebrafish (Gering and Patient, 2005; Lawson et al., 2002), but similar to observations in the mouse (Coultas et al., 2010), VEGFA does not lie downstream of HH, as expression of *Vegfa* in the somites and *Dll4* (as a readout for VEGFA activity) in the DA was not affected in cyclopamine-treated embryos (Figure 7C). We conclude that there is no epistatic relationship between ETO2, ETV6, and the HH pathway or VEGFA and HH at this stage of development in *Xenopus*. Therefore, ETO2 and ETV6 play distinct, essential, and unique roles in the regulation of *Vegfa* and the programming of HSCs.

## Discussion

Three sites of VEGFA production related to hematopoiesis have been described in developing *Xenopus* embryos: DLP mesoderm, somites, and hypochord. The first two regions express all three VEGFA isoforms, whereas the hypochord predominantly expresses the short isoform (Cleaver and Krieg, 1998). Full depletion of VEGFA prevents hemangioblast specification, DA precursor migration, and DA formation (Ciau-Uitz et al., 2010b). Interestingly, depletion of the TF ETV6 in early embryogenesis specifically inhibits VEGFA expression from the DLP and somites. This affects correct programming of the adult hemangioblasts, arterial specification, and, consequently, HSC formation, through both paracrine and autocrine mechanisms (Ciau-Uitz et al., 2010b). It does not prevent migration of the DA precursors to the midline and vessel formation (Ciau-Uitz et al., 2010b), these events being supported by expression of the short, diffusible VEGFA isoform in the hypochord (Cleaver and Krieg, 1998) (Figure 7D).

Our current study provides insight into VEGFA functions. Through downregulation and isoform-specific knockdown of VEGFA, we have uncovered an extrinsic role for VEGFA in establishing the HSC program that is independent of hemangioblasts specification, vessel formation, and arterial development. We have also further dissected the requirements for VEGFA in DA development, prior to HSC specification. Our study has therefore uncoupled the requirement for VEGFA signaling in establishing the endothelial, arterial, and HSC programs in DA precursors (Figure 7D).

Although a role for VEGFA_122_ in the hypochord for the migration and coalescence of the DA progenitors has been suggested (Cleaver and Krieg, 1998), contribution of the other VEGFA isoforms toward specification of the DA/HSC program was unknown. Through the combined analyses of ETO2 morphant embryos, *Vegfa* hypomorphs, and *Vegfa* isoform-specific morphants, we have unveiled a specific requirement for VEGFA medium and/or long isoform and assigned additional specific functions to the short isoform (Figure 7D). First, signaling from VEGFA_170_ in the somites instructs the HSC program of the DA/HSC progenitors as they migrate toward the midline in a paracrine manner. Whether this function is specific to VEGFA_170_ or shared by both VEGFA_170_ and VEGFA_190_ could not be addressed because of toxicity issues experienced with exogenous *Vegfa*_*190*_ mRNA. This signaling is critically required at later stages for development of the hemogenic endothelium in the newly formed DA. Second, VEGFA_122_ not only directs migration of the DA precursors (Cleaver and Krieg, 1998) but is also sufficient for hemangioblasts specification, lumenization, and arterialization. Separately, our findings suggest additional functions for the two anchored VEGFA isoforms in vasculogenesis, such as in the development of the PCV and the trunk vasculature; this, however, remains to be fully investigated.

A dose-dependent regulation by VEGFA was initially revealed in haploinsufficient, embryonic lethal *Vegfa* mouse models displaying blood vessel defects (Carmeliet et al., 1996; Ferrara et al., 1996). Using the embryonic stem cell differentiation model, Lanner et al. (2007) reported that VEGFA regulates endothelial development and arterial specification in a graded manner, with high levels being absolutely required to induce the arterial program. Extending these observations, we now show that partial downregulation of VEGFA (in *Vegfa* hypomorphs) disturbs the arterial but not the endothelial program of the DA. As VEGFA_170_ and VEFGA_190_ are not required for this process (see *Vegfa* isoform-specific morphants), this strongly suggests the existence of a threshold of VEGFA_122_ short signaling to fully support the arterial program.

The fact that the VEGFA_122_ short isoform does not rescue the HSC defects in the ETO2 morphants suggests the necessity of contacts between somitic cells/ECM and DA precursors. The differential functional importance of the medium/long and short VEGFA isoforms is consistent with previous findings. First, it was recently shown that matrix-bound medium isoform differentially activates FLK1 receptor when compared to soluble VEGFA isoform (through recruitment of different receptor partners and differential phosphorylation), therefore triggering different intracellular responses (Chen et al., 2010). Second, Zhang et al. (2008) highlight the differential effects of short and medium VEGFA isoforms on MEK/ERK and Src pathways. Third, mouse VEGFA medium isoform specifically interacts with the coreceptor NRP1, triggering formation of a specific ternary complex (VEGFA/NRP1/FLK1) that modulates subsequent bioactivity in endothelial cells (Soker et al., 2002). Therefore, we propose that binding of VEGFA_170_ (and/or VEGFA_190_) to its receptors on the DA precursors elicits a downstream response different from that triggered by the soluble (VEGFA_122_) isoform. Investigating the intracellular molecular cascade triggered by VEGFA_170_/VEGFA_190_ will help understand how the HSC program is initiated.

In contrast to HSC specification, development of the endothelial and arterial programs does not seem to require direct contacts between DA precursors and VEGFA-producing cells but exposure to soluble VEGFA_122_. Our study provides a platform to investigate the specific mechanisms engaged by VEGFA short isoform in these processes and to fully assess the functional differences between the different VEGFA isoforms.

VEGFA is known to function upstream of the NOTCH pathway in arterial and HSC development. However, the mechanisms by which VEGFA regulates this pathway are not clear. Demonstration of the functional importance of NOTCH often results from studies using inhibitors of the intracellular canonical pathway that is shared by all NOTCH receptors. It is therefore difficult to assess which NOTCH is functionally important in a specific cellular pathway. As detailed below, our data suggest that different isoforms of VEGFA are successively required upstream of two distinct NOTCH receptors (NOTCH1 and NOTCH4, the only two receptors expressed in the DA [A.C.-U., and R.P., unpublished data]) and clarify their role in HSC programming.

(1) The first molecular defect observed in the DA of *Eto2* mutant embryos is the absence of expression of *Notch1.* We propose that high levels of medium and/or long somitic VEGFA are necessary for the initiation of *Notch1* expression in the DA and therefore provide evidence for a mechanistic link between VEGFA and NOTCH1 in HSC specification. Moreover, as in the mouse, NOTCH1 appears to be the “hematopoietic” NOTCH in *Xenopus.*
(2) Together with the previously established function of VEGFA in arterial specification (Ciau-Uitz et al., 2010b; Gering and Patient, 2005; Lawson et al., 2002), our data demonstrate that high levels of VEGFA_122_ in the somites and/or in the DLP are sufficient to trigger the arterial program. We propose that this may be initiated through NOTCH4 signaling. Indeed, in wild-type embryos, *Notch4* is first detected at stage 31 and *Notch1* at stage 34, after the onset of the arterial program (A.C.-U. and R.P., unpublished data). Moreover, both *Notch4* and *Dll4* (the arterial-specific NOTCH ligand [Duarte et al., 2004]) are expressed in the DA of *Eto2*-morphant embryos from stages 32 to 39.
(3) *Gata2*, the early hematopoietic target of the NOTCH pathway (Robert-Moreno et al., 2005), is expressed at stage 32 in the DA of ETO2-depleted embryos, but, in contrast to the arterial markers, its expression is gradually downregulated thereafter and absent by stage 39. It is also absent in the VEGFA hypomorphs and isoform-specific morphants. Extending previous observations (Robert-Moreno et al., 2005), our data therefore suggest that *Gata2* expression is initiated by NOTCH4, and, unlike the arterial genes, requires NOTCH1 for its maintenance.

In conclusion, distinct isoforms of VEGFA sequentially activate the arterial and blood programs. NOTCH4 appears critical for specification of the arterial program and initiation of *Gata2* expression. NOTCH1 appears critical for maintaining *Gata2* expression and triggering HSC specification, very likely by initiating *Runx1* expression (Burns et al., 2005; Gering and Patient, 2005). A distinct, non-cell-autonomous requirement for the NOTCH ligands deltaC and deltaD in the establishment of definitive hematopoiesis was recently described in somites in zebrafish (Clements et al., 2011), highlighting increasing evidence for the role of the somites in HSC specification and stressing the complexity of NOTCH inputs in this process.

Our study demonstrates that ETO2 functions in a non-cell-autonomous manner in the somites during *Xenopus* development and controls one of the major pathways required for HSC production, illustrating the multifunctionality of TFs. Indeed, in mammals, ETO2 has cell autonomous functions in adult mouse HSCs. Therefore, our study provides an example of a TF employing different mechanisms of action in distinct cellular settings and stages of development. The TF ETV6 is another example of this, as we recently demonstrated (Ciau-Uitz et al., 2010b). Mechanistically, ETO2, like other members of the ETO protein family, acts as a corepressor (Schuh et al., 2005 and references therein) and is therefore unlikely to directly control *Vegfa* transcription. We propose that ETO2 may repress a repressor of *Vegfa* in the somites. Alternatively, ETO2 could control *Vegfa* levels in an isoform-specific manner in the somites through regulation of a splicing regulator.

In conclusion, our study has uncovered a critical, non-cell-autonomous role for ETO2 in establishing the adult hematopoietic system in developing *Xenopus* embryos. This function is mediated by the nondiffusible isoform(s) of VEGFA that is required for *Notch1* expression in HSC precursors. These findings further our mechanistic understanding of how major signaling pathways control key stages of blood development and, by doing so, may help design protocols for in vitro production of HSCs with regenerative and therapeutic purposes.

## Experimental Procedures

### Morpholino Design

*Eto2* ATG MO (*Eto2*-MO, 5′-ATCAGCCGGTGAGTCTGGCATTGTA-3′) and *Eto2* 5′UTR MO (*Eto2*-MO2 5′-CAATGGTCCCAGCAGAAGTAGATC-3′) were designed. *Vegfa* MOs have been previously described (Kälin et al., 2007). The VEGFA isoform-specific MO was designed to target the intron6/exon7 splice junction (Vegfa-MOi6e7 5′-GACTGCAAAAAGCAAAATGATGACA-3′); the control MO targets the exon6/intron7 junction (MOc 5′-ATTGGATTTGAGCAAGCATAAGCGC-3′). All MOs were obtained from Gene Tools (Corvallis, OR, USA). Titrations of the MOs were carried out to determine the optimal concentration for knockdown of gene expression without detrimental effects to general embryonic development. We initially tested 40/50/80 ng of *Eto2*-MO. Embryos injected with 80 ng were morphologically abnormal from stage 39 onward. Embryos were thereafter injected with 48/50 ng of *Eto2*-MO with no obvious abnormalities. Regarding *Eto2*-MO2, there were no obvious abnormalities, and the hematopoietic defects were observed at 25/40/50 ng of MO; embryos were routinely injected with 25/30 ng. Finally, embryos were routinely injected with 12.5 ng of Vegf-MO and 25 ng of Vegfa-MOi6e7.

### *X. laevis* Embryos

*Xenopus* embryos were obtained and cultured as previously described (Smith and Slack, 1983; Walmsley et al., 1994) and staged according to Nieuwkoop and Faber (1967). Embryos were injected at the two-cell stage as previously described (Walmsley et al., 1994). For the somite-hypochord dissections, tissues were dissected out from stage 27 WT and *Eto2*-MO embryos. mRNA was extracted from somite-hypochord regions or whole embryos with Trizol (Invitrogen, Carlsbad, CA, USA); complementary DNA synthesis was carried out with Superscript III (Invitrogen).

### Synthesis of mRNA for Injection

The Gateway cloning system (Invitrogen) was used to obtain an ETO2:GFP fusion construct, where green fluorescent protein (GFP) was fused to a 225 bp fragment of the *Eto2* 5′ sequences containing the initiation codon. The expression constructs containing *Vegfa* sequences were a kind gift of Prof. Paul Krieg, University of Arizona. mRNAs were prepared using the Ambion mMESSAGE machine kit (Austin, TX, USA). Embryos were injected with 2, 3, or 4 ng of RNA.

### In Situ Hybridization

Whole-mount in situ hybridization (WMISH) and in situ hybridization on sections (ISHS) were performed as previously described (Ciau-Uitz et al., 2000; Walmsley et al., 1994). For probe synthesis details, see the Supplemental Experimental Procedures. The *Xenopus Eto* probe was a kind gift from Dr. Koyano-Nakagawa, University of Minnesota.

## Supplemental Information

Document S1. Figures S1–S6 and Supplemental Experimental Procedures
