# Supplementary material for: Uncoupling VEGFA Functions in Arteriogenesis and Hematopoietic Stem Cell Specification
Source: Dev Cell. 2013 Jan 28;24(2):144–58. doi: 10.1016/j.devcel.2012.12.004 (PMC3560039; doi:10.1016/j.devcel.2012.12.004)
Supplement: Document S1. Figures S1–S6 and Supplemental Experimental Procedures [file mmc1.pdf]

Developmental Cell, Volume *24*

Supplemental Information

Uncoupling VEGFA Functions in Arteriogenesis and Hematopoietic Stem Cell Specification

Amy Leung, Aldo Ciau-Uitz, Philip Pinheiro, Rui Monteiro, Jie Zuo, Paresh Vyas, Roger Patient, and Catherine Porcher

Supplemental Information Inventory

Figure S1. Design and characterisation of *Eto2* morpholinos, related to Figure 1.

Alignment of Eto2 and Eto-related sequences in the regions targeted by the MOs. Sequence and in vivo characterisation of Eto2 MOs.

Figure S2. Analysis of arteriogenesis and vasculogenesis in *Eto2*-MO and *Eto2*-MO2 injected embryos, related to Figures 2, 3.

Analysis of vasculogenesis in Eto2 morphants confirms normal endothelialisation and arterialisation.

Figure S3. *Eto2* is not expressed in the DA - Expression pattern of *Eto2*-related transcripts during development, related to Figure 4.

Expression of Eto2-related transcripts does not overlap with that of Eto2 in hematopoietic tissues.

Figure S4. Zebrafish ETO2 is required for HSC emergence, related to Figures 1-4.

The zebrafish studies provide an independent confirmation of the phenotype observed in Xenopus.

Figure S5. Expression of a panel of markers implicated in the development of the DA/HSC progenitors in stage 27 WT and Eto2 morphant embryos, related to Figure 5.

Investigation of potential early defects that may be involved in the Eto2 morphant hematopoietic phenotype.

Figure S6. *Vegfa* hypomorph embryos and *Vegfa*-MOi6e7 morphants phenocopy the *Eto2* morphants, related to Figure 6.

Extended analysis of vasculogenesis and arteriogenesis in Vegfa morphants.

Figure legends

Supplemental Experimental Procedures

Supplemental References

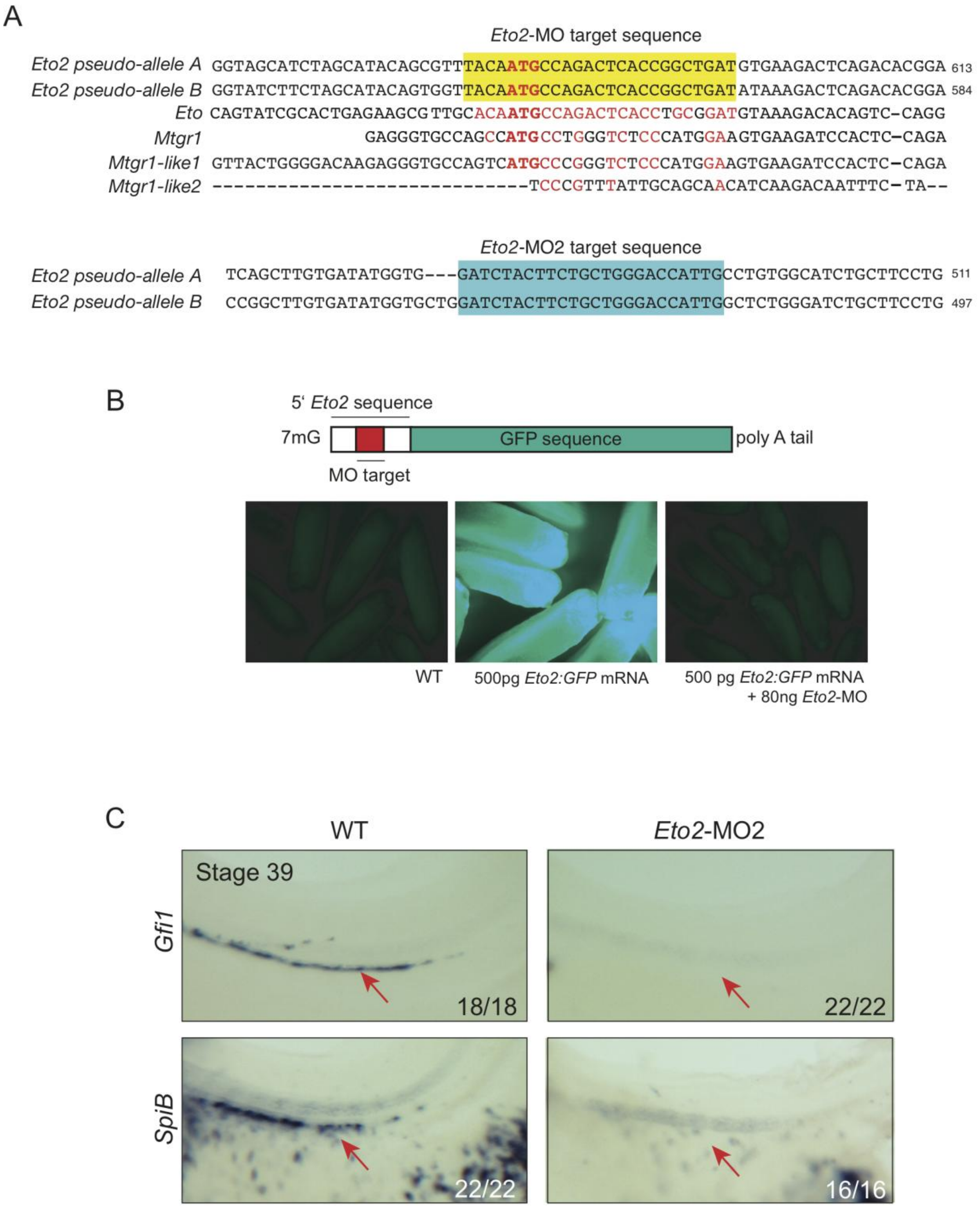
A
Eto2-MO target sequence
Eto2 pseudo-allele A GGTAGCATCTAGCATACAGCGTTTACAATGCCAGACTCACCGGCTGATGTGAAGACTCAGACACGGA 613
Eto2 pseudo-allele B GGTATCTTCTAGCATACAGTGGTTACAATGCCAGACTCACCGGCTGATATAAAGACTCAGACACGGA 584
Eto CAGTATCGCACTGAGAAGCGTTGCACAATGCCAGACTCACCTGCGGATGTAAAGACACAGTC-CAGG
Mtgr1 GAGGGTGCCAGCCATGCCTGGGTCTCCCATGGAAGTGAAGATCCACTC-CAGA
Mtgr1-like1 GTTACTGGGGACAAGAGGGTGCCAGTCATGCCCGGGTCTCCCATGGAAGTGAAGATCCACTC-CAGA
Mtgr1-like2 -----------------------------TCCCGTTTATTGCAGCAACATCAAGACAATTTC-TA--
Eto2-MO2 target sequence
Eto2 pseudo-allele A TCAGCTTGTGATATGGTG---GATCTACTTCTGCTGGGACCATTGCCTGTGGCATCTGCTTCCTG 511
Eto2 pseudo-allele B CCGGCTTGTGATATGGTGCTGGATCTACTTCTGCTGGGACCATTGGCTCTGGGATCTGCTTCCTG 497
B
5‘ *Eto2* sequence
7mG
GFP sequence
poly A tail
MO target
WT
500pg *Eto2:GFP* mRNA
500 pg *Eto2:GFP* mRNA + 80ng *Eto2*-MO
C
WT
Eto2-MO2
Stage 39
Gfi1
18/18
22/22
SpiB
22/22
16/16

Leung_Figure S2, related to Figures 2, 3

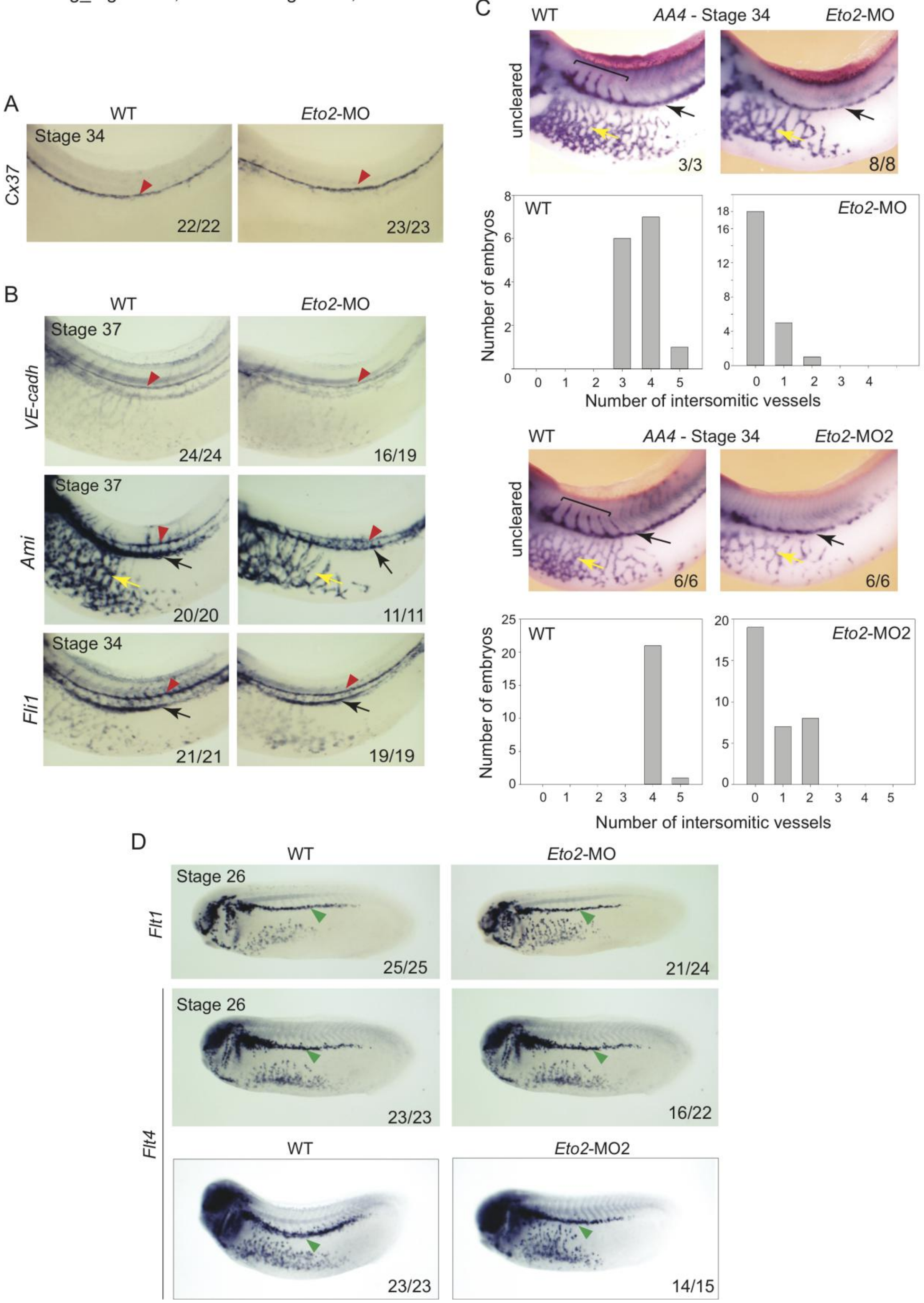

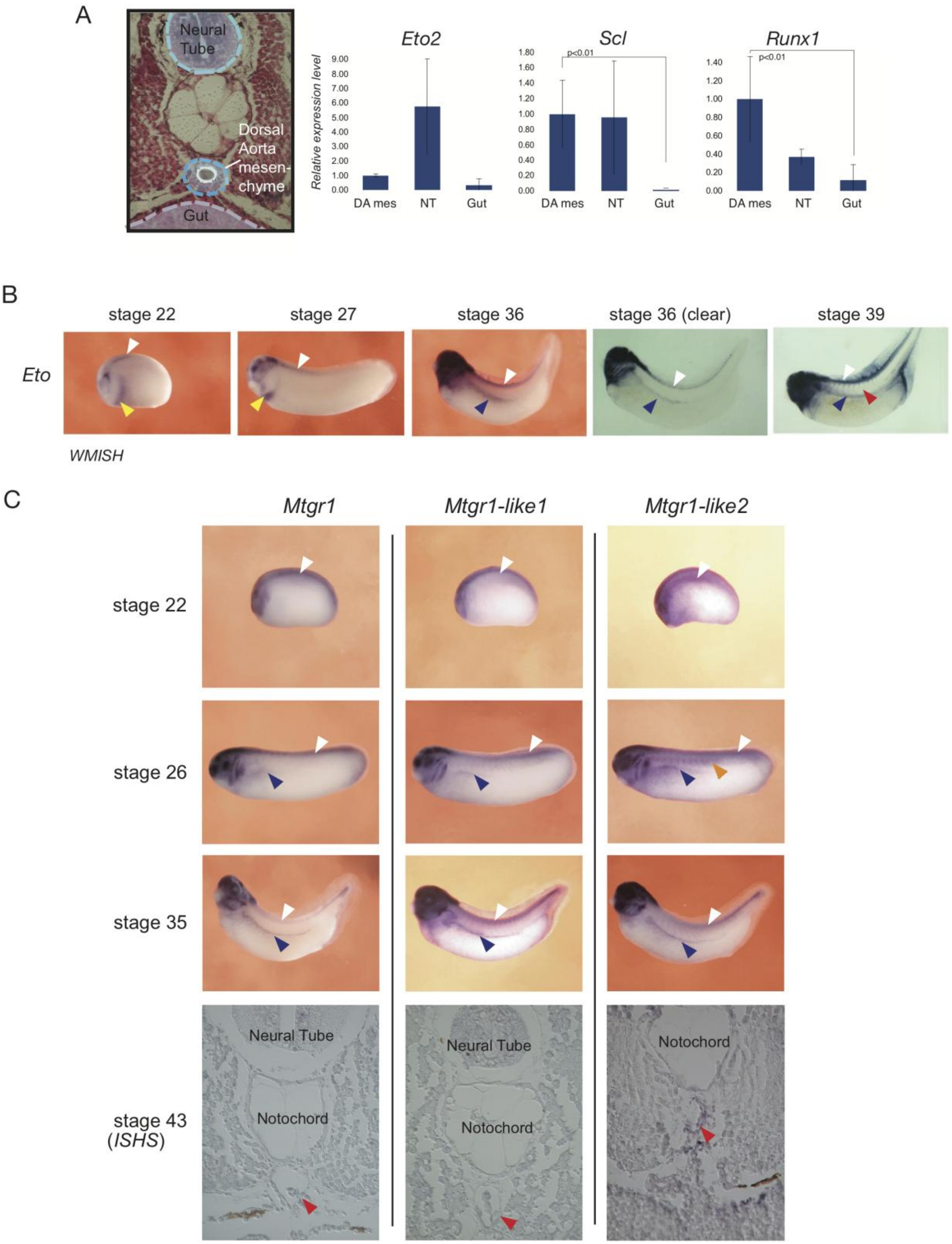
A
Neural Tube
Dorsal Aorta mesen-chyme
Gut
Eto2
Scl
Runx1
Relative expression level
p<0.01
p<0.01
DA mes
NT
Gut
B
stage 22
stage 27
stage 36
stage 36 (clear)
stage 39
Eto
WMISH
C
Mtgr1
Mtgr1-like1
Mtgr1-like2
stage 22
stage 26
stage 35
stage 43 (ISHS)
Neural Tube
Notochord

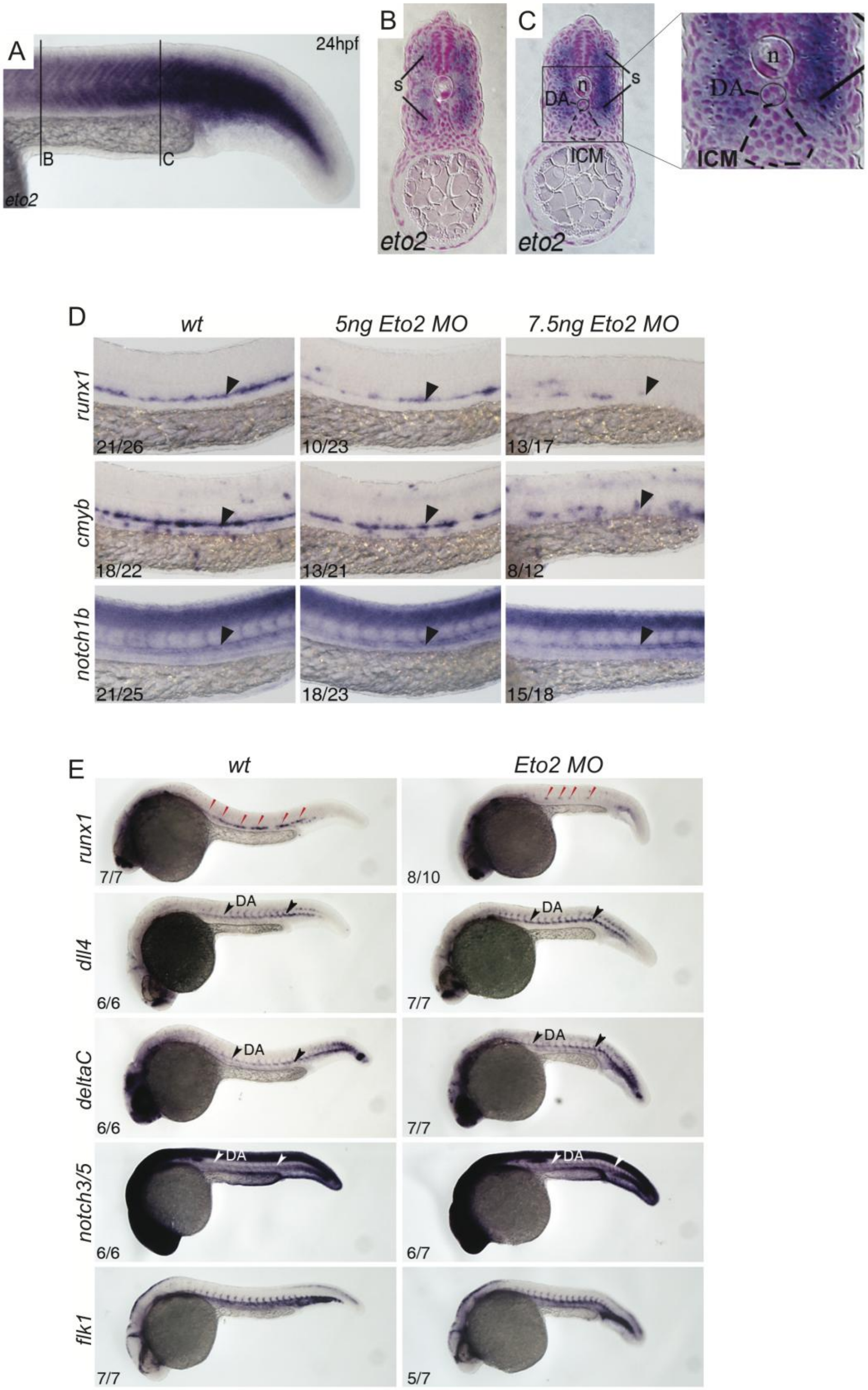
A
24hpf
B
C
eto2
B
s
eto2
C
n
DA
s
ICM
eto2
n
DA
ICM
D
wt
5ng Eto2 MO
7.5ng Eto2 MO
runx1
21/26
10/23
13/17
cmyb
18/22
13/21
8/12
notch1b
21/25
18/23
15/18
E
wt
Eto2 MO
runx1
7/7
8/10
dll4
DA
6/6
7/7
deltaC
6/6
7/7
notch3/5
6/6
6/7
flk1
7/7
5/7

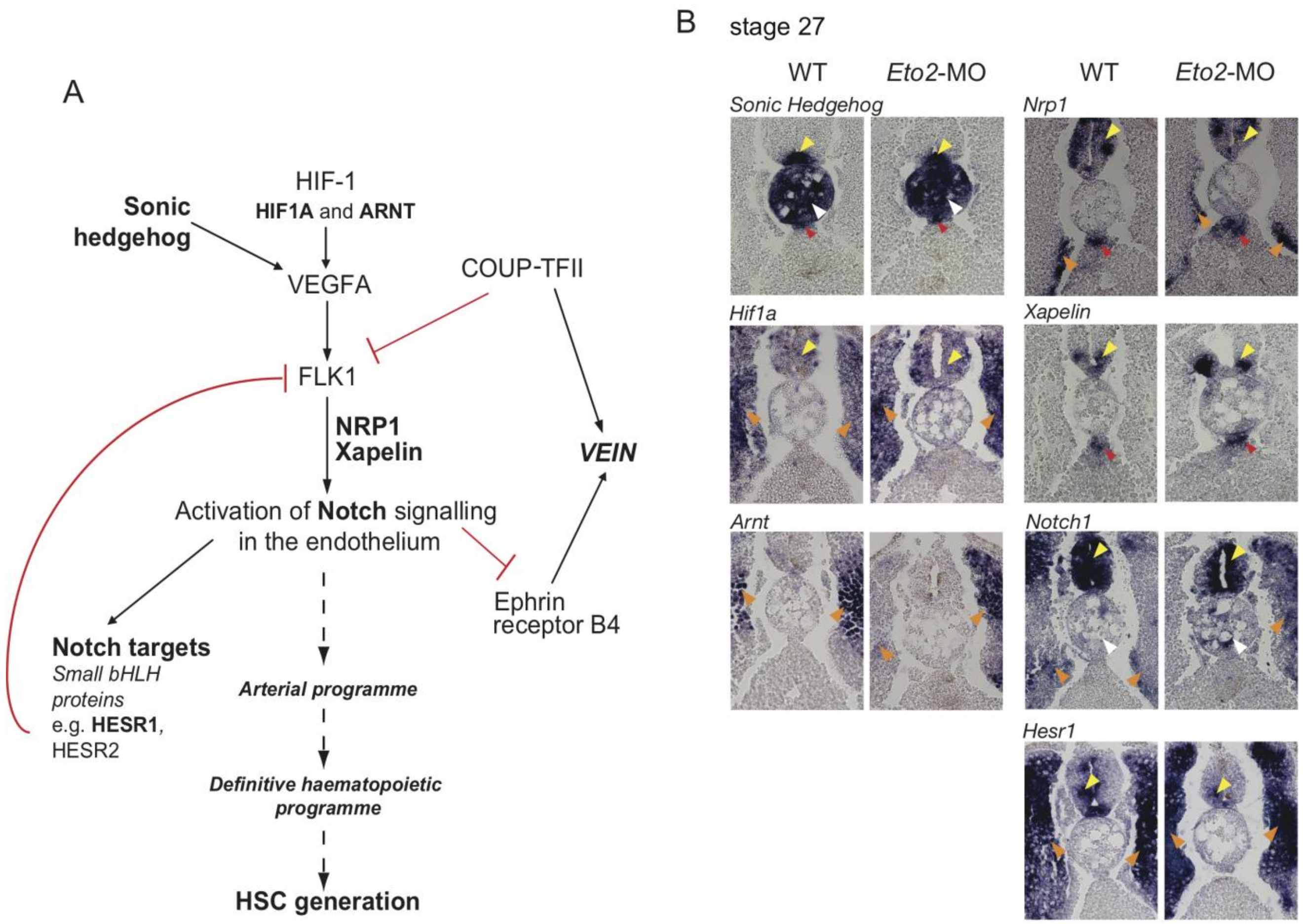
A
HIF-1
HIF1A and ARNT
Sonic hedgehog
VEGFA
COUP-TFII
FLK1
NRP1
Xapelin
VEIN
Activation of Notch signalling in the endothelium
Ephrin receptor B4
Notch targets
Small bHLH proteins
e.g. HESR1, HESR2
Arterial programme
Definitive haematopoietic programme
HSC generation
B
stage 27
WT
Eto2-MO
Sonic Hedgehog
Nrp1
Hif1a
Xapelin
Arnt
Notch1
Hesr1

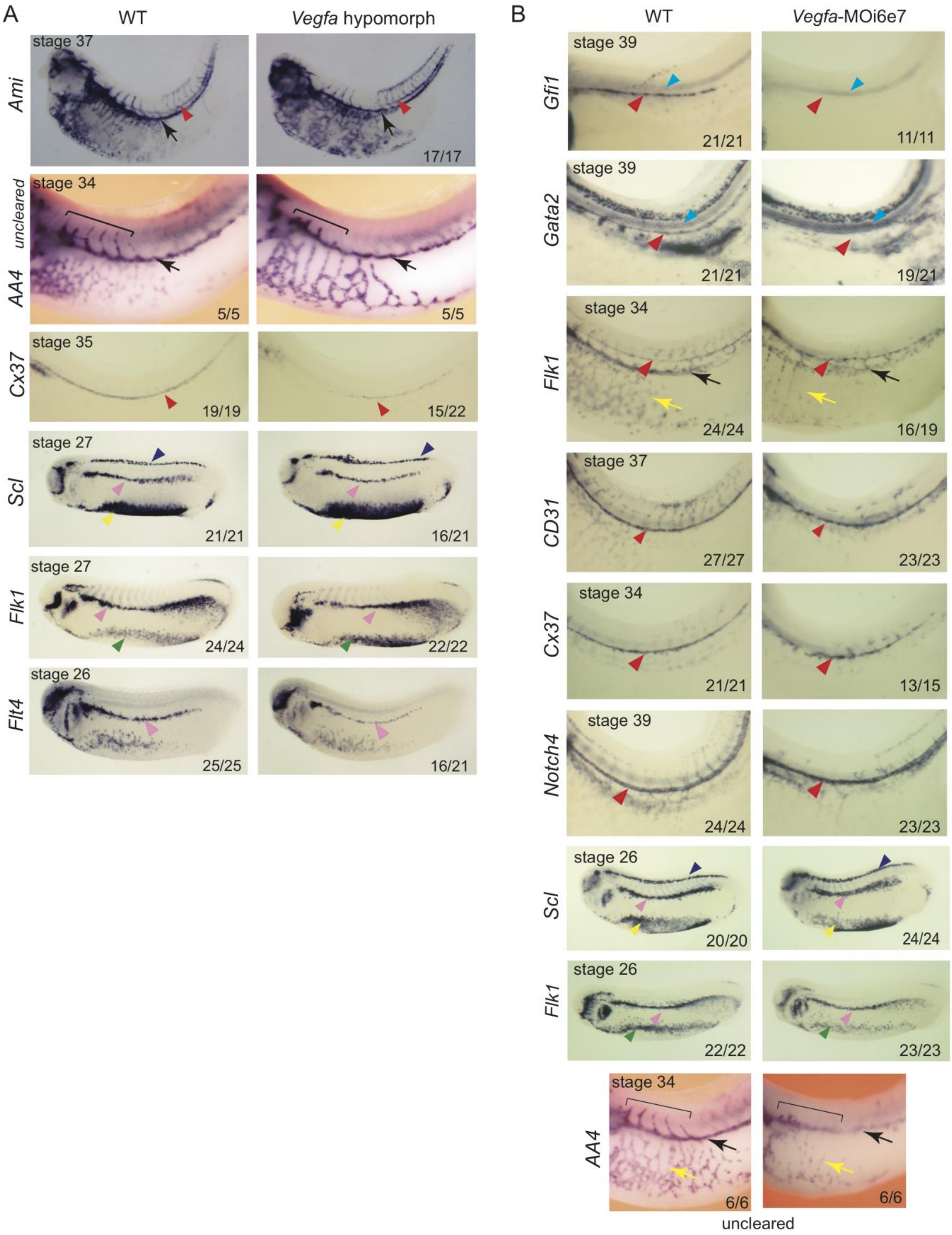
A
WT
Vegfa hypomorph
stage 37
Ami
17/17
stage 34
uncleared
AA4
5/5
5/5
stage 35
Cx37
19/19
15/22
stage 27
Scl
21/21
16/21
stage 27
Flk1
24/24
22/22
stage 26
Flt4
25/25
16/21
B
WT
Vegfa-MOi6e7
stage 39
Gfi1
21/21
11/11
stage 39
Gata2
21/21
19/21
stage 34
Flk1
24/24
16/19
stage 37
CD31
27/27
23/23
stage 34
Cx37
21/21
13/15
stage 39
Notch4
24/24
23/23
stage 26
Scl
20/20
24/24
stage 26
Flk1
22/22
23/23
stage 34
AA4
6/6
6/6
uncleared

Figure Legends

Figure S1, related to Figure 1. Design and testing of the *Eto2* morpholinos (MOs)

(A) Xenopus laevis has a pseudo-tetraploid genome; genes are therefore present in two “pseudo-allele” forms. Sequence alignment of the beginning of the coding sequence of *Eto2* pseudo-alleles A and B and *Eto*-related genes (top) and of the 5’ UTR of *Eto2* pseudo-alleles A and B (bottom); the target regions for the “ATG” MO (ETO2-MO) and “5’UTR” MO (ETO2-MO2) are highlighted in yellow and blue respectively. The MOs were designed to have 100% homology to the mRNAs transcribed from both *Eto2* pseudo-alleles. The nucleotides in red are conserved between *Eto2* and the *Eto*-related transcripts in the region targeted by *Eto2*-MO.

The *Eto*-related transcripts (*Eto, Mtgr1* and two transcripts with high similarity to *Mtgr1*, namely *Mtgr1-like1* (MGC68858) and *Mtgr1-like2* (IMAGE 5156021), accession numbers are in Supplemental Experimental Procedures, section Probes used for in situ hybridisaiton) are not targeted by the *Eto2* MOs. For *Mtgr1*, *Mtgr1-like1* and *Mtgr1-like2*, the sequences targeted by the MOs are sufficiently divergent to avoid unspecific targeting effects. Of note, 5 mismatches are recommended to avoid unspecific targeting effects (Eisen and Smith 2008). Given that only 3 nucleotides differ between the *Eto* sequence and the sequence recognised by *Eto2*-MO, the *Eto* transcripts could potentially be targeted. However, as detailed in Figure S3, they are not expressed in hematopoietic or somitic tissues and are therefore unlikely to have any function in hematopoiesis. The sequences targeted by *Eto2*-MO2 are too divergent to be aligned; this MO is therefore very unlikely to interfere with translation of any of the *Eto*-related mRNAs.

(B) A GFP-reporter mRNA tethered to the 5’ region of ETO2 containing the *Eto2*-MO target sequence (*Eto2*:GFP) was used to test the efficacy of the MO in vivo. Embryos co-injected with *Eto2*-MO and *Eto2:*GFP mRNA had no visible GFP expression (right panel), as opposed to mRNA alone (middle panel). *Eto2*-MO is therefore able to bind to its intended target sequence *in vivo* and to block translation.

(C) Expression analysis of the HSC markers *Gfi1* and *SpiB* in *Eto2*-MO2 injected embryos. Red arrow; DA. Numbers at the bottom of the panels indicate the number of embryos with the given phenotype out of the total number examined. Whole mounts are shown with anterior to the left and dorsal to the top.

Figure S2, related to Figures 2,3. Analysis of arteriogenesis and vasculogenesis in *Eto2*-MO and *Eto2*-MO2 injected embryos

Expression analysis of the arterial marker *Cx37* (**A,** stage 34), the endothelial genes *VE-cadh, Ami, Fli1* and *AA4* (**B-C**, stages 37/34), *Flt1 and Flt4* (**D**, stage 26) in *Eto2*-MO and *Eto2*-MO2 morphants. Red arrowhead, DA; black arrow, PCV; yellow arrow, trunk vasculature; green arrowhead, DLP. **(C)** Uncleared embryos, bracket indicates intersomitic vessels (ISVs) sprouting from the PCV in wild-type embryos; the graphs show the number of ISVs observed in wild-type, *Eto2*-MO and *Eto2*-MO2 embryos. Numbers at the bottom of the panels indicate the number of embryos with the given phenotype out of the total number examined. Whole mounts are shown with anterior to the left, dorsal to the top.

Figure S3. Related to Figure 4. Eto2 is not expressed in the DA - Expression pattern of *Eto2*-related transcripts during development

(A) Laser Capture Microdissection (LCM) was used to isolate specific tissues from stage 39 embryonic sections; the DA and surrounding mesenchyme (DA mes), the neural tube (NT) and gut. NT tissues were intended as a positive control for amplification of the *Eto2* transcripts and the gut as a negative control (as expected from WMISH and ISHS results, Figure 4). Gene expression analysis was carried out for *Eto2* and the known HSC markers *Scl* and *Runx1* by Reverse Transcription Real Time PCR. Results were normalised to ODC. Errors bars represent the standard deviation from two independent experiments. Unlike the HSC markers, *Eto2* expression in the DA mesenchyme is not significantly different from that observed in gut when compared to the high levels observed in the NT.

(B, C) Expression of *Eto*, *Mtgr1*, *Mtgr1-like1* and *Mtgr1-like2* was examined during *Xenopus* development **(B)** Expression of *Eto* is detected in the heart fields (stage 22, yellow arrowhead), in the heart (stage 27, yellow arrowhead), in neural cells (stage 22; neural tube at stages 27, 36, 39, white arrowheads) and in the PCV (stages 36/39, blue arrowhead). There was no expression in the DA at stage 39 (red arrowhead). **(C)** Expression of *Mtgr1*, *Mtgr1-like1* and *Mtgr1-like2* is observed in neural cells (white arrowheads stage 22; neural tube at stages 26 and 35), and in the PCV (initiates anteriorly at stage 26, is established by stage 35; blue arrowheads). *Mtgr1-like2* is expressed at low levels in the somites (stage 26, orange arrowhead). ISHS at stage 43 shows no expression of *Mtgr1* and *Mtgr1-like1* in the DA (red arrowhead) and a faint staining for *Mtgr-like2* in the region of the DA and surrounding mesenchyme. However, given the lack of homology in the sequences targeted by the *Eto2* MOs, it is highly unlikely that expression of *Mtgr-like2* was affected in the knock-down experiments.

Whole mounts are shown with anterior to the left and dorsal to the top. Sections are in transverse orientation with dorsal to the top.

Figure S4, related to Figures 1-4. Zebrafish ETO2 is required for HSC emergence

(A-C) As in *Xenopus*, *Eto2* is expressed in the trunk somites **(A)** but not in the dorsal aorta (DA), as seen in transverse sections along the trunk **(B, C)**, at 24hpf (hours post-fertilisation)**;** s, somites; ICM, intermediate cell mass. **(D)** To test the function of ETO2 in hematopoietic development, we knocked-down its expression using a morpholino that targets the zebrafish *Eto2* (Meier et al. 2006). A dose-dependent loss of the HSC markers *runx1* and *cmyb* (another HSC marker (Murayama et al. 2006)) in the DA (black arrowheads) was observed with increasing amounts of *Eto2* MO, at 28hpf. In contrast, expression of the arterial marker *notch1b* was unaffected. **(E)** At 28hpf, none of the arterial markers analysed (*dll4*, *dlC*, *notch3/5;* DA, black and white arrowheads (Lawson and Weinstein 2002; Nicoli et al. 2008; Rowlinson and Gering 2010)) was affected in *Eto2* morphants, whereas *runx1* expression was severely downregulated (red arrowheads). *Flk1* expression is grossly normal in *Eto2* morphants confirming normal endothelialisation. Therefore, down-regulation of *Eto2* in zebrafish embryos leads to non cell-autonomous phenotypic defects that are very similar to those observed in *Xenopus Eto2* morphant embryos.

Figure S5, related to Figure 5. Expression of a panel of markers implicated in the development of the DA/HSC progenitors in stage 27 WT and *Eto2* morphant embryos.

(A) Schematic diagram detailing the signalling events that are proposed to be involved in the specification of the DA and the HSC program; adapted from (Diez et al. 2007).

(B) Expression of selected markers was examined in stage 27 WT and *Eto2* morphant embryo sections by ISHS. There was no discernible alteration in the expression level or the pattern of these markers in the *Eto2* morphants.

Hypochord; red arrowheads, Neural tube; yellow arrowheads, Somites; orange arrowheads, Notochord; white arrowheads. Sections are in transverse orientation with dorsal to the top.

Figure S6, related to Figure 6. *Vegfa* hypomorph embryos and *Vegfa*-MOi6e7 morphants phenocopy the *Eto2* morphants

(A) Expression of endothelial (*Ami, AA4, Flt4*), arterial (*Cx37*) and hemangioblast (*Scl* and *Flk1*) genes was examined in *Vegfa* hypomorph embryos, by WMISH at the stages indicated. Note that *AA4* expression was examined on uncleared embryos.

(B) *Vegfa* medium/long isoform morphants (*Vegfa*-MOi6e7) recapitulate the *Eto2* morphant phenotype in the DA. WMISH analysis of hematopoietic (*Gfi1, Gata2*) endothelial (*Flk1, CD31*), arterial (*Cx37* and *Notch4*) and hemangioblast (*Scl, Flk1*) markers reveals that endothelialisation, arterialisation and

hematopoietic specification do take place in the morphant DA (red arrowheads). Note, however, the absence of ISVs (*AA4*, uncleared embryos, brackets), the weak staining of the PCVs (black arrows) and the limited development of the trunk vasculature (yellow arrows) in the *Vegfa*-MOi6e7 morphants. Numbers at the bottom of the panels indicate the number of embryos with the given phenotype out of the total number examined. Whole mounts are shown with anterior to the left and dorsal to the top.

Red arrowheads, DA; black arrows, PCV; brackets, ISVs; pink arrowheads, DLP; yellow arrowheads, VBI; dark blue arrowheads, *Scl* expression in the neural tube; green arrowheads, *Flk1* expression in the trunk endothelium; light blue arrowheads, notochord; yellow arrows, trunk vasculature. Numbers at the bottom of the panels indicate the number of embryos with the given phenotype out of the total number examined. Whole mounts are shown with anterior to the left and dorsal to the top.

Supplemental Experimental Procedures

Laser Capture Microdissection (LCM)

Xenopus embryos were washed in 30% sucrose:OCT and 100% OCT. Individual embryos were then transferred and orientated in a cryostat mould filled with OCT and snap-frozen. 20µm sections (20-30 sections from the DA region; ~ 10 sections from gut and NT regions) were cut from the blocks in a cryostat and transferred to Membraneslides (Leica). The slides were stained with Toluidine Blue (0.1%) before microdissection. RNA was extracted from pooled micro-dissected samples using a QIAGEN RNeasy micro kit.

Real-Time quantitative PCR

Primers for both TAQMAN and SYBRGREEN PCR were designed using the Primer Express Software Version 3.0 program (Applied Biosystems). Details of primers used are below. PCR reactions were performed using TaqMan® Universal PCR Master Mix or SYBR® GREEN PCR Master Mix (Applied Biosystems). All results were normalised to levels of ornithine decarboxylase (ODC).

Real-Time and standard PCR primers

**SYBRGreen Primers**		
XLVEGFA122_F	CAACATCACCATGCAGATAATGAA	**Detection of Xenopus VEGFA$_{122}$**
XLVEGFA122_R	CGTGGCTTTTCACATTTTTCCT	
XLVEGFA170ALL_F	AATCATTGTGAGCCTTGCACAG	**Detection of Xenopus VEGFA$_{170}$**
XLVEGFA170ALL_R	GGCTTTTCACATCTGCAAGTCC	
XLVEGFA190_F	CGAGGGAAGGGCCTTAAACG	**Detection of Xenopus VEGFA$_{190}$**
XLVEGFA170ALL_R	GGCTTTTCACATCTGCAAGTCC	
**TaqMan Primers**		
XLETO2-F	CAGAAAGCTGTGTCGGAAGCAG	**Detection of Xenopus XLETO2**
XLETO2-R	TGATGCTTGGCGTTTTGCCTC	
XLETO2-Probe	FAM-ACGATCTCATCACTTTGGAACGCACCAAG-TAMRA	
XSCL-F	CCATGCTCTATGGGCTCAATC	**Detection of Xenopus SCL**
XSCL-R	AAGGTGTCTGGGTCACCAAAGT	*Walmsley et al, Blood, 2007*
XSCL-Probe	FAM-CCCCTGGCGTCAGATAACAGTGGC-TAMRA	
Xrunx1-F	GGATCCTACCACCAGTTCTCTAT	**Detection of Xenopus Runx1**
Xrunx1-R	CCCGTGGAAGCGTTTGTG	*Walmsley et al, Blood, 2007*
Xrunx1-Probe	FAM-ATCTCCGCCTCGCATCCTCCCA-TAMRA	
XODC-F	CTGCCGCCTCAGTGTGAAXODC-F	**Detection of Xenopus ODC**
XODC-R	GCAGCCACTGCCAACATG	*Walmsley et al, Blood, 2007*
XODC-Probe	FAM-ACCCTTAAAACAAGCAGGCTGCTTCTGGA-TAMRA	

VegfA FL Fwd: ccccttaaccggaaaatcat (at ATG)
VegfA FL Rev: tgtccctcttcttggaatgc (at 3'UTR) designed using BC169428, 793bp PCR fragment

Fish maintenance and morpholino injections

Fish were bred, maintained and staged as described (Westerfield 2007). A morpholino oligonucleotide (MO) was used to target zebrafish *Eto2* (Meier et al. 2006).

In situ hybridization, sections and image acquisition (zebrafish)

Whole mount hybridization was carried out as described (Jowett and Yan 1996). An EST containing the zebrafish *Eto2* cDNA (Acession number: CK693846, Imagenes, Germany) was used as a template to generate an *Eto2* probe. DIG-labelled antisense RNA probes were transcribed from linearized templates using T3, T7 or Sp6 RNA polymerases (Roche, Burgess Hill, United Kingdom). Embryos were bleached and prepared for whole mount imaging as described (Monteiro et al. 2011); alternatively, embryos were embedded in JB-4 resin (Electron Microscopy Sciences) and sectioned according to the manufacturer's protocol. Sections were counterstained with neutral red and mounted in Pertex (Leica).

Whole mount photography was done on a Nikon DXM 1200 digital camera and Nikon ACT-1 software (version 2.12) mounted on a Nikon SMZ 1500 zoom stereomicroscope (Nikon, Melville, NY); sections were imaged on a Nikon DMX1200C camera and Nikon Elements software mounted on a Eclipse E600 microscope.

Probes used for *in situ* hybridisation

Gene Name	EST ID	Accession Number	Restriction Enzyme	RNA Polymerase	Reference
**Xenopus probes**					
Runx1			SalI	T7	Tracey et al., 1998
Gata2			XbaI	SP6	Walmsley et al., 1994
VegfA			BamHI	T7	Cleaver et al., 1997
Scl			XhoI	SP6	Ciau-Uitz et al., 2010
Flk1	NIBB XL087o23	BJ092634	NotI or SacI or SmaI	T7	Ciau-Uitz et al., 2010
αT4-globin			EcoRI	SP6	Walmsley et al., 1994
Eto2	NIBB XL185m19	BJ635188	NotI or BamHI	T7	This report
Eto2	IMAGE 5130002	BX852558	SalI or SmaI	T7	This report
Eto			EcoRI	T7	Koyano-Nakagawa & Kintner, 2005
MTGR1	IMAGE 5571316	BC044006	EcoRI	T7	This report
MTGR1-like1	IMAGE 4680246	BC057713	SalI or EcoRI or EcoRV	T7	This report
MTGR1-like2	IMAGE 5156021	CA790039	SalI or EcoRI or EcoRV	T7	This report
SpiB	IMAGE 5537169	AAH46671	SalI or EcoRI	T7	Ciau-Uitz et al., 2010
Lmo2	IMAGE 4174203	AAH97502	SalI or SmaI or EcoRI	T7	Ciau-Uitz et al., 2010
Gfi1	IMAGE 8547327	EB645267	EcoRI or ClaI	T7	Ciau-Uitz et al., 2010
Notch4	IMAGE 4684242	BQ735158	SalI or SmaI	T7	Ciau-Uitz et al., 2010
Dll4	IMAGE 7876232	DT435811	EcoRI or ClaI or BamHI	T7	Ciau-Uitz et al., 2010
EphrinB2a	IMAGE 4724740	BC057724	SalI or SmaI	T7	Ciau-Uitz et al., 2010

Notch1	NIBB XL056a06	BJ087286	NotI or SacI	T7	This report
Hesr1	IMAGE 7020309	BC084410	SmaI or EcoRI	T7	This report
Ami	IMAGE 7204180	CK797755	EcoRV or EcoRI or SmaI	T7	This report
Tie2	NIBB XL064i22	BJ092234	NotI or SacI or SmaI	T7	Ciau-Uitz et al., 2010
Cx37	IMAGE 5512615	BC073347	SalI or smaI or EcoRI	T7	This report
Vecad	IMAGE 7638101	CX408264	SalI or ClaI	T7	This report
AA4	IMAGE 5515354	CF290542	SalI or SmaI	T7	This report
Flt1	IMAGE 4959298	AAH56023	SalI or SmaI	T7	Ciau-Uitz et al., 2010
Flt4	IMAGE 4970772	CF289873	SalI	T7	Ciau-Uitz et al., 2010
Etv6	NIBB XL153n17		NotI or SacI	T7	Ciau-Uitz et al., 2010
Ptc1			EcoRI	SP6	Takabatake et al., 2001
HIF1α	IMAGE 4930371	BC043769	SmaI or EcoRI	T7	This report
Arnt/HIF1β	NIBB XL032m15	BJ056716	NotI	T7	This report
Shh	NIBB XL096o20	BJ089257	NotI or SacI or SmaI	T7	This report
Nrp1	IMAGE 4969039	BX844937	SalI or EcoRI or EcoRV	T7	This report
Apelin	IMAGE 6631163	BU906199	SalI or SmaI or EcoRI	T7	This report
**Zebrafish probes**					
Eto2			CK693846	See Extended Experimental Procedures	
Runx1			HindIII	T7	Kalev-Zylinska et al., 2002
cMyb			EcoRI	T7	Thompson et al 1998
Notch1b	IMAGE 3725324	AI793882	HindIII	T7	This report
Dll4			SpeI	T7	Rowlinson and Gering, 2010
DeltaC			XbaI	T7	Rowlinson and Gering, 2010
Notch3			PstI	T7	Gering and Patient, 2005
Flk1			EcoRI	T7	Fouquet et al, 1997

Supplemental References

Diez H, Fischer A, Winkler A, Hu CJ, Hatzopoulos AK, Breier G, Gessler M. 2007. Hypoxia-mediated activation of Dll4-Notch-Hey2 signaling in endothelial progenitor cells and adoption of arterial cell fate. *Exp Cell Res* **313**: 1-9.

Eisen JS, Smith JC. 2008. Controlling morpholino experiments: don't stop making antisense. *Development* **135**: 1735-1743.

Jowett T, Yan YL. 1996. Double fluorescent in situ hybridization to zebrafish embryos. *Trends Genet* **12**: 387-389.

Lawson ND, Weinstein BM. 2002. In vivo imaging of embryonic vascular development using transgenic zebrafish. *Dev Biol* **248**: 307-318.

Monteiro R, Pouget C, Patient R. 2011. The gata1/pu.1 lineage fate paradigm varies between blood populations and is modulated by tif1gamma. *Embo J* **30**: 1093-1103.

Murayama E, Kissa K, Zapata A, Mordelet E, Briolat V, Lin HF, Handin RI, Herbomel P. 2006. Tracing hematopoietic precursor migration to successive hematopoietic organs during zebrafish development. *Immunity* **25**: 963-975.

Nicoli S, Tobia C, Gualandi L, De Sena G, Presta M. 2008. Calcitonin receptor-like receptor guides arterial differentiation in zebrafish. *Blood* **111**: 4965-4972.

Nowak DG, Woolard J, Amin EM, Konopatskaya O, Saleem MA, Churchill AJ, Ladomery MR, Harper SJ, Bates DO. 2008. Expression of pro- and anti-angiogenic isoforms of VEGF is differentially regulated by splicing and growth factors. *J Cell Sci* **121**: 3487-3495.

Rowlinson JM, Gering M. 2010. Hey2 acts upstream of Notch in hematopoietic stem cell specification in zebrafish embryos. *Blood* **116**: 2046-2056.

Westerfield M. 2007. THE ZEBRAFISH BOOK: A guide for the laboratory use of zebrafish (Danio rerio). *5th Edition edn (Eugene, University of Oregon Press)*.

Xu J, Dou T, Liu C, Fu M, Huang Y, Gu S, Zhou Y, Xie Y. 2011. The evolution of alternative splicing exons in vascular endothelial growth factor A. *Gene* **487**: 143-150.
